# Artificial intelligence‐based motion tracking in cancer radiotherapy: A review

**DOI:** 10.1002/acm2.14500

**Published:** 2024-08-28

**Authors:** Elahheh Salari, Jing Wang, Jacob Frank Wynne, Chih‐Wei Chang, Yizhou Wu, Xiaofeng Yang

**Affiliations:** ^1^ Department of Radiation Oncology Emory University Atlanta Georgia USA; ^2^ Radiation Oncology Icahn School of Medicine at Mount Sinai New York New York USA; ^3^ School of Electrical and Computer Engineering Georgia Institute of Technology Atlanta Georgia USA

**Keywords:** artificial intelligence, intrafraction motion, motion management, radiotherapy

## Abstract

Radiotherapy aims to deliver a prescribed dose to the tumor while sparing neighboring organs at risk (OARs). Increasingly complex treatment techniques such as volumetric modulated arc therapy (VMAT), stereotactic radiosurgery (SRS), stereotactic body radiotherapy (SBRT), and proton therapy have been developed to deliver doses more precisely to the target. While such technologies have improved dose delivery, the implementation of intra‐fraction motion management to verify tumor position at the time of treatment has become increasingly relevant. Artificial intelligence (AI) has recently demonstrated great potential for real‐time tracking of tumors during treatment. However, AI‐based motion management faces several challenges, including bias in training data, poor transparency, difficult data collection, complex workflows and quality assurance, and limited sample sizes. This review presents the AI algorithms used for chest, abdomen, and pelvic tumor motion management/tracking for radiotherapy and provides a literature summary on the topic. We will also discuss the limitations of these AI‐based studies and propose potential improvements.

## 1 INTRODUCTION

Radiotherapy aims to deliver a high dose of radiation to treatment targets while minimizing the dose to surrounding healthy tissues. The advent of flattening filter‐free (FFF) treatment delivery brought higher dose rate beams and greater normal tissue sparing due to the sharp dose fall‐off outside the tumor.^1^, ^2^ The FFF technique has widened the therapeutic window, ushering in new radiation delivery techniques such as SRS and SBRT.^2^, ^3^ Intrafraction motion monitoring is particularly needed for the SRS and SBRT, where a high dose is delivered to the target in a few fractions, and narrow margins are needed to spare healthy tissues.^4^ This treatment technique is commonly implemented in the lung, abdomen, and sometimes pelvis, where the efficiency of the treatment can be significantly reduced due to intrafraction respiratory, cardiac, gastrointestinal, and urinary motion during the treatment.^5^, ^6^, ^7^ Internal organ movement may cause underdosing or overdosing of targets or normal tissues, potentially causing treatment failure and increasing normal tissue toxicity.^8^, ^9^, ^10^ In this setting, real‐time tumor tracking techniques are essential to localize targets and ensure accurate treatment delivery without compromising treatment quality due to motion.  Conventional motion management techniques include: using a dynamic multileaf collimator (MLC) to optimize MLC positions based on target motion,^11^ adjusting the radiation beam and robotic couch according to target movement,^12^ implanting electromagnetic transponders in the soft tissue to localize tumors or placing transponders on the body surface to monitor the motion,^13^, ^14^ utilizing stereoscopic kilovoltage (kV) imaging in conjunction with a six‐degrees‐of‐freedom couch,^15^ employing an optical surface tracking system using an infrared camera to automatically align patients by tracking infrared (IR) markers on their skin or a rigid template,^16^ and using ultrasound (US) guidance equipped with a hardware device to hold a US probe in a position that maintains the target within the US imaging field of view during the treatment session.^17^ Recently, magnetic resonance imaging (MRI) integrated with the linear accelerator has been developed for monitoring intrafraction motion during dose delivery.^18^ The purpose of this review is not to provide a detailed explanation of each of the techniques as mentioned earlier, interested readers are directed to Wu et al.,^19^ which contains additional information.

Though various methods for intrafraction motion management have been developed,^19^ direct detection of the target during the treatment is often not feasible.^20^ Alternatively, indirect tumor localization facilitated with artificial intelligence (AI) approaches can be used. After decades of development, modern AI approaches can be categorized into classic machine learning (ML) and deep learning (DL). When applied to motion management, these techniques have successfully analyzed medical images and made motion predictions. AI‐based techniques can be applied to several disease sites as well as many imaging modalities including MRI, computed tomography (CT), and US. Due to the wide range of possible applications, numerous motion‐tracking strategies have been proposed. Recently, ML approaches integrating radiomics have been developed to analyze medical images. Radiomics is a novel topic in the field of radiology, which extracts mineable quantitative features from medical images. The extracted features contain information on size, shape, and texture from the region of interest and can be used to develop ML models to predict the target position.^20^, ^21^ Traditional algorithms, including artificial neural network (ANN),^22^ support vector machine (SVM),^23^ light gradient boosting machine (LightGBM),^21^ decision trees (DT),^20^ random forests (RF),^24^ have been used for predicting tumor position.

In addition to classic ML approaches, many authors have employed DL for the real‐time tracking of tumors. Convolutional neural network (CNN) is one of the backbone DL architectures used in various medical image/object recognition and classification.^25^, ^26^, ^27^ It employs principles of linear algebra, specifically convolution operations, to extract features and recognize patterns within images. Along with CNN, U‐Net and generative adversarial networks (GAN) have been used for motion management. U‐Net is a vital semantic segmentation framework of CNN that performs effectively for pixel‐level prediction tasks.^28^ GAN is an ML model where two neural networks compete against each other to create more accurate predictions. Other more advanced networks such as recurrent neural networks (RNN),^29^ region convolutional neural networks (R‐CNN),^30^ Siamese networks,^31^ you‐only‐look‐once (YOLO),^32^ long short‐term memory (LSTM),^33^ and encoder‐decoder networks^34^ have also been introduced for real‐time tumor tracking.

This work aims to review AI‐based approaches for tumor tracking in the thoracic, abdominal, and pelvic regions, discuss present limitations, and provide potential solutions for more accurate outcomes.

## 2 LITERATURE SEARCH

**FIGURE 1:**
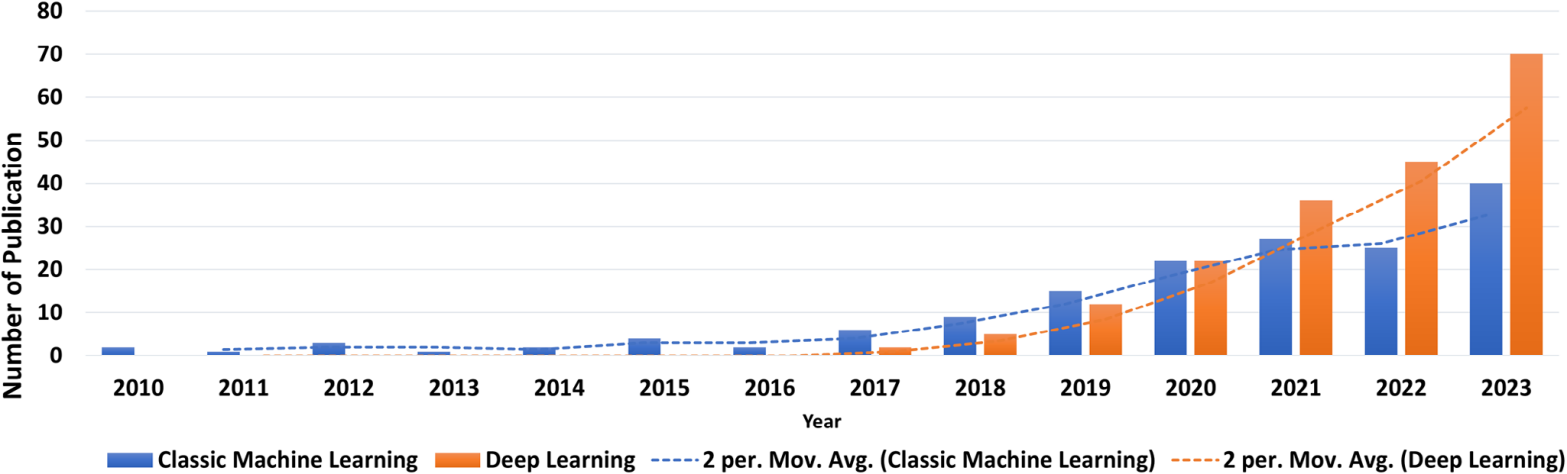
Number of publications using classic ML and DL‐based motion management since 2010. Deep learning has exponentially increased since 2017. The number of publications in 2023 is an estimation based on the number of publications from January to August 2023.

**FIGURE 2:**
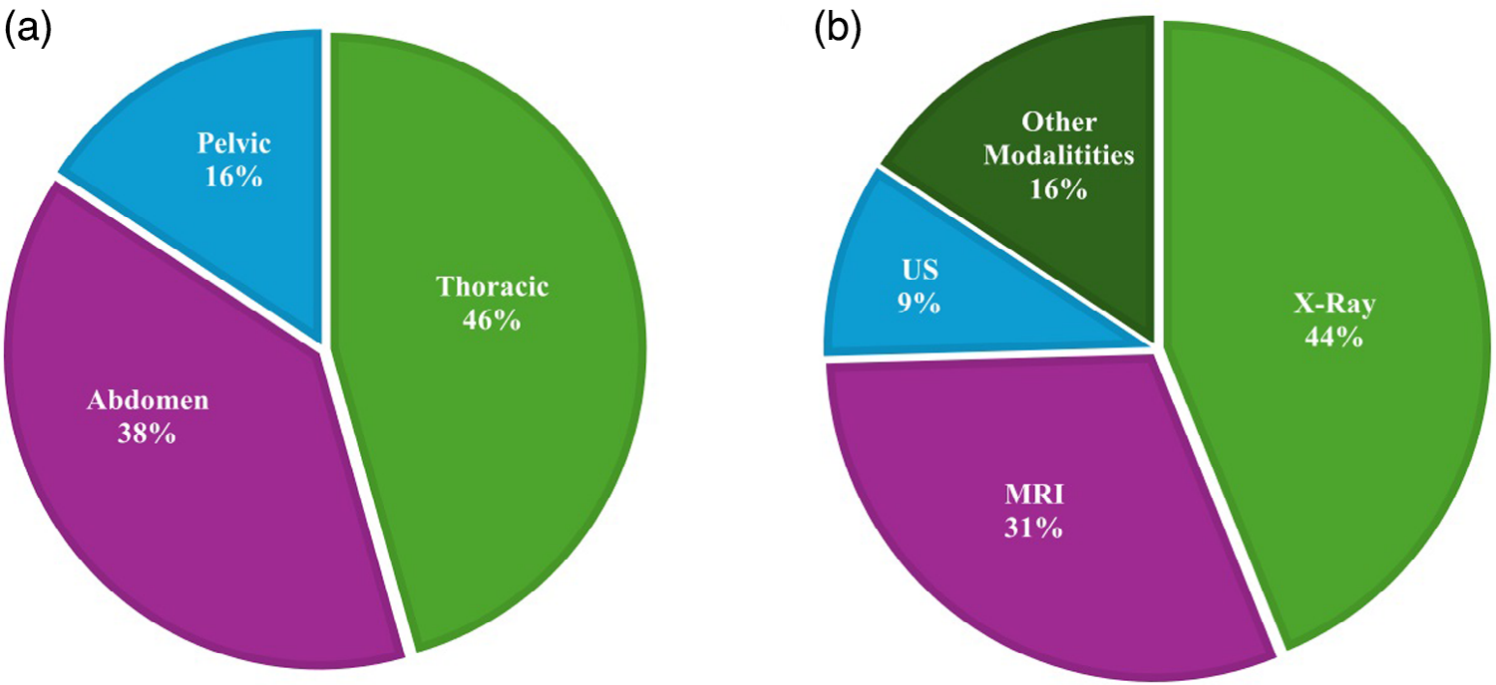
Statistical analysis of artificial intelligence‐based target tracking in PubMed (www.pubmed.gov) since 2005. (a) The percentage of studies conducted on each treatment site. (b) The percentage of each modality in AI‐based motion tracking. X‐ray includes CT, CBCT, 4DCT, MV, and kV fluoroscopic images. Other modalities include respiratory gating, electromagnetic transponders, and optical surface monitoring. (c) The percentage of classic ML versus DL models.

**FIGURE 3:**
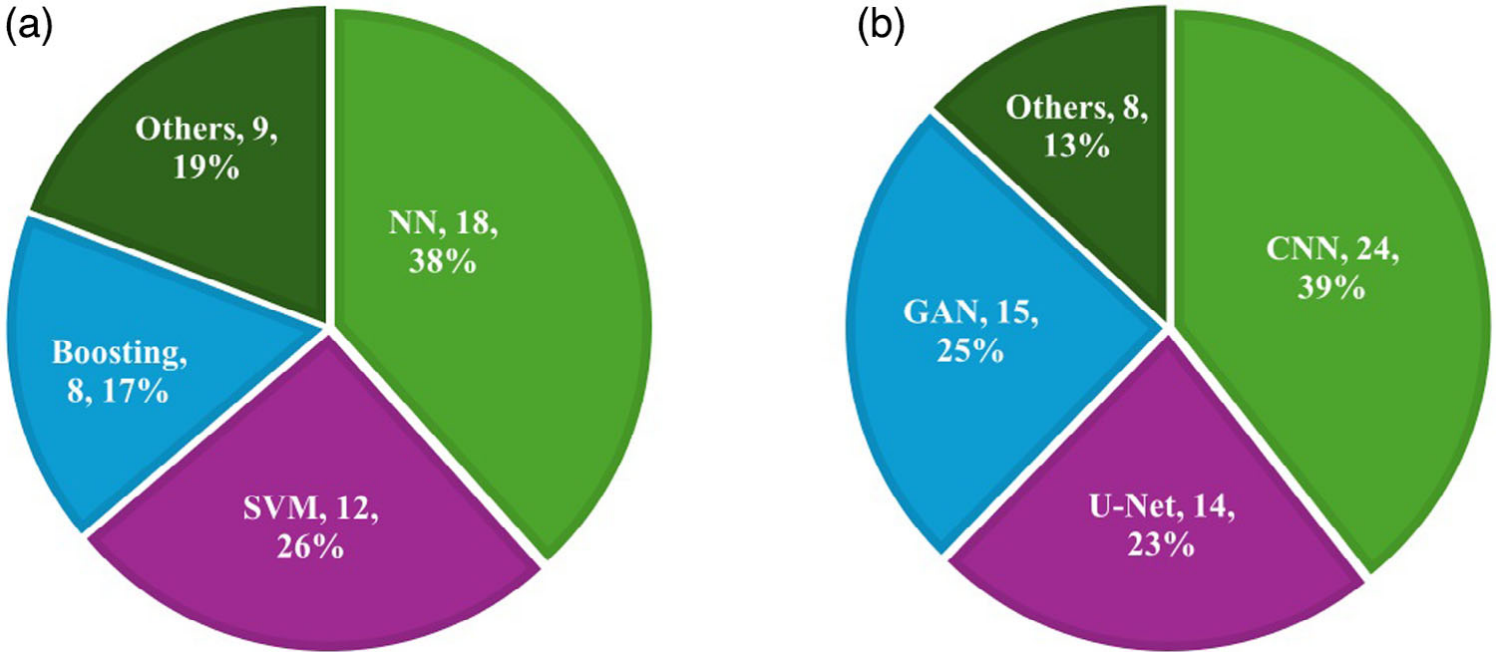
Statistical analysis of artificial intelligence‐based target tracking in PubMed (www.pubmed.gov) since 2005. (a) The percentage of papers using different classic machine learning algorithms. (b) The percentage of different deep learning models. CNN, convolutional neural network; GAN, generative adversarial network; NN, neural network; SVM, support vector machine.

This review focuses on intrafraction motion management using classic ML and DL algorithms. To ensure this systematic review is valuable to users, we followed the Preferred Reporting Items for systematic reviews and meta‐analyses (PRISMA).^35^ For this aim, we only considered peer‐reviewed papers as they undergo an evaluation process where journal editors and experts critically assess the article's quality and scientific merit. In this regard, the PubMed search engine was used with a time window from January 2005 to August 2023. The search keywords were limited to “cancer or radiotherapy,” “deep learning or machine learning or artificial intelligence,” “imaging or image‐guided radiation therapy or IGRT,” and “motion.” The initial search yielded 183 records. However, after excluding literature reviews, and publications not related to human studies only 91 papers remained. Moreover, a citation search was conducted on other literature resulting in an additional 17 papers; therefore, a total of 108 articles were included in this review study. Figure 1 shows the surging number of yearly peer‐reviewed publications containing the terms “artificial intelligence/machine learning/deep learning,” “motion,” “radiotherapy/cancer,” and “images” from 2010 to April 2024 in the PubMed database (www.pubmed.gov). Based on the available information, the number of publications in 2024 is just a linear extrapolation from the number of papers in the first quarter to the remainder of the year. Figure 2 displays the percentage of studies for each treatment site and imaging modality, respectively. In Figure 2, x‐ray includes CT, CBCT, 4DCT, MV, and kV fluoroscopic images. Other modalities refer to respiratory gating, electromagnetic transponders, and optical surface monitoring. Figure 3 shows the results of the statistical analysis of the included research studies.

## 3 MOTION MANAGEMENT FOR VARIOUS TREATMENT SITES

### 3.1 Thoracis

According to the American Cancer Society, lung cancer is the leading cause of cancer‐related death in the United States.^36^ Thus, lung cancer has been extensively studied. Radiotherapy is a standard of care in the multi‐disciplinary treatment of lung cancer and is being increasingly used, but it is challenged by intrafraction motion during treatment. Intrafraction motion is primarily a result of respiration, with a lesser contribution of the cardiac cycle to tissue displacement and deformation. Respiration induces organ motion and anatomical shifts, which can significantly reduce the accuracy of dose delivery, causing failure of tumor control or normal tissue injury.^37^ Respiratory gating and breath‐hold are common solutions to manage target motion,^38^ but both techniques provide only a limited representation of the complex respiratory motion pattern.^39^ Several AI methods across several imaging modalities have been developed to address these problems. Table 1 summarizes AI‐based target tracking using different image modalities, including details on the dataset (e.g., the number of patients), publication year, AI model, input, image modality, and key findings in thoracic regions. As we can see in Table 1, the majority of authors used x‐rays (e.g., CT, CBCT, 4DCT, kV X‐ray) in the chest area. CT scans are considered the preferred imaging modality for examining tissue abnormalities in the chest because of their superior efficacy, especially when compared to MRI. This is because the chest cavity consists of pleural cavities (lungs and pleura), which contain a significant amount of air. The presence of air can reduce the accuracy and sensitivity of MRI, which is a functional imaging technique that relies on water diffusivity.^40^ CT scan, on the other hand, is based on x‐ray photon absorption, which is inversely proportional to the density of the object. Therefore, it can precisely detect lesions in the thoracic regions due to sharp differences in tissue density.

**TABLE 1:** Summary of publication using artificial intelligence for real‐time thoracic tumor tracking.

Author	Year	AI model	Image modality	No. samples	Key findings in results
Isaksson et al.^41^	2005	Linear filters, adaptive linear filters, and adaptive neural networks (Feed‐forward neural networks)	kV x‐ray	3 (1 pancreatic, 2 lung cancer patients)	The neural network (NN) achieved better tracking accuracy than other algorithms. The NN was able to predict the position of the tumor up to 800 ms in advance. In one case, the linear filter was completely unable to predict the tumor position (nRSME = 100%).
Kakar et al.^42^	2005	Adaptive neuro‐fuzzy inference system (ANFIS)	Infrared camera	11 lung cancer patients	Root‐mean‐square error (RMSE) for coached patients was 6% and for non‐coached patients (breath freely) was 35% over an interval of 20 s.
Murphy and Dieterich^43^	2006	The linear adaptive filter and the adaptive nonlinear neural network	Optical tracking system	9 lung cancer patients	Predict respiratory signals up to 1 s in advance. The neural network outperformed the linear filter. In some cases, the linear filter was completely unable to adapt to the breathing signal
Yan et al.^44^	2006	ANN	The signal of a simulator and IR camera	4 patients	The target position can be predicted using external surrogates if the correlation between internal target motion and external marker signals is consistent.
Zhang et al.^45^	2007	Principal component analysis (PCA)	CT	4 lung cancer patients	The average discrepancies between the predicted model and ground truth were 1.1 ± 0.6 mm LR, 1.8 ± 1.0 mm AP, and 1.6 ± 1.4 mm SI.
Cui et al.^23^	2008	SVM	kV x‐ray	5 lung cancer patients	The SVM can predict the gating signal to deliver the dose to the target with tumor coverage greater than 90%.
Lin et al.^46^	2009	ANN, SVM	kV x‐ray	9 lung cancer patients (ten fluoroscopic image sequences)	ANN approach is more accurate than SVM in terms of classification accuracy and recall rate. The ANN achieved higher accuracy than SVM (96.3 ± 1.6 vs. 94.9 ± 1.7). The average running time for ANN is less than SVM (6.7 ms vs. 11 ms).
Lin et al.^47^	2009	Linear regression (LR), two‐degree polynomial regression, ANN, and SVM	kV x‐ray	10 lung cancer patients	Two‐degree polynomial regression tends to be overfitted. ANN performs better and is more robust than the other models. ANN achieved the lowest localization errors within all models.
Riaz et al.^48^	2009	Multidimensional adaptive filter, SVM	Optical tracking system	14 lung cancer patients	The SVM prediction results are more accurate than the other model with the RSME equal to 1.26. The RMSE of the multidimensional adaptive filter was 1.71.
Torshabi et al.^49^	2010	ANN and Fuzzy logic	Cyberknife Synchrony	15 chest cases and 4 abdominal cases (10 worse cases, 10 control cases)	The best performance was obtained using Fuzzy logic algorithms. The result of ANN was comparable to Cyberknife Synchrony and the calculation time of ANN was higher than other models. The error reduction with respect to Synchrony®, measured at the 95% confidence level is 10.8% for the fuzzy logic approach and 8.7% for ANN.
Cervino et al.^50^	2011	ANN, template matching algorithm with surrogate tracking using the diaphragm	MRI	5 healthy volunteers	The matching approach detects the target position more accurately than the ANN model.
Krauss et al.^51^	2011	LR, NN, kernel density estimation, and SVM	kV x‐ray	12 breathing data	When considering all sampling rates and latencies, the observed prediction errors normalized to errors of using no prediction for NN, SVR, LR, and KDE were 0.44, 0.46, 0.49, and 0.55, respectively.
Li et al.^52^	2011	PCA	4DCT	Two phantoms and 11 image sets from eight patients.	The modeling error was within 0.7 ± 0.1 mm. The mean 3D error was 1.8 ± 0.3 mm.
Fayad et al.^53^	2012	PCA	4DCT and synchronized RPM signal	10 lung cancer patients	The model is substantially accurate when it includes both phase and amplitude data with the model error of 1.35 ± 0.21 mm.
Yun et al.^22^	2012	ANN (feed‐forward neural network)	MRI (MR‐linac)	29 lung cancer patients	For 120–520 ms system delays, mean RMSE values of 0.5−0.9 mm (ranges 0.0−2.8 mm from 29 patients) were observed.
Torshabi et al.^54^	2014	ANFIS	Cyberknife Synchrony	10 (lung and pancreas cancer patients)	The ANFIS model was able to decrease tumor tracking errors significantly compared with the ground truth database.
Li et al.^55^	2015	MLR	4DCT	11 lung cancer patients (2 scans for each patient so 22 4DCT in total)	The ML model was able to predict the diaphragm motion with acceptable error (0.2 ± 1.6 mm).
W Bukhari and S‐M Hong^56^	2015	An extended Kalman filter (LCM‐EKF) to predict the respiratory motion and a model‐free Gaussian process regression (GPR) to correct the error of the LCM‐EKF prediction.	Gating system	31 patients (304 traces of respiratory motion)	This model reduced the root‐mean‐square error by 37%, 39%, and 42% for a duty cycle of 80% at lookahead lengths of 192 ms, 384 ms, and 576 ms respectively compared to the non‐correction method.
Yun et al.^57^	2015	ANN	MRI (MR‐linac)	One phantom and four lung cancer patients	The phantom study yields a mean Dice similarity index (DSI) of 0.95−0.96, and a mean Hausdorff distance (HD) of 2.61−2.82 mm. The mean DSI of 0.87−0.92, with a mean HD of 3.12−4.35 mm for the patient study.
Bukovsky et al.^58^	2015	Multilayer perceptron (MLP), Quadratic Neural Unit (QNU)	3D time series of lung motion	356 simulations for QNU and 2475 for MLP	The mean absolute error was 0.987 mm and 1.034‐1.041 mm for QNU and MLP respectively. The QNU model provided better results with a mean absolute error of 0.987 and was faster than MLP.
Park et al.^59^	2016	Fuzzy deep learning (FDL), CNN, Hybrid motion estimation based on extended Kalman filter (HEKF)	CyberKnife Synchrony	130 lung cancer patients	FDL showed fewer variations than CNN and HEKF. The RMSE of FDL was 30 % better than CNN and HEKF. The average computing time using a central processing unit (CPU) for FDL, CNN, and HEKF was 1.54 ± 5.01 ms, 254.32 ± 11.68 ms, and 253.56 ± 10.74 ms respectively.
Teo et al.^60^	2018	A 3‐layer perceptron neural network	MV images	47 (27 in group 1 and 20 in group 2, unseen data)	The average MAE (group 1) = 0.59 ± 0.13 mm, and the average MAE (group 2) = 0.56 ± 0.18 mm. The average RMSE (group 1) = 0.76 ± 0.34 mm and for group 2 was 0.63 ± 0.36 mm.
Terunuma et al.^61^	2018	CNN	3DCT or 4DCT	4 lung cancer patients	The proposed method achieved accurate tumor tracking of low visibility tumors of over 0.95 based on the Jaccard index, and accurate tumor tracking with an error of approximately 1 mm.
Edmunds et al.^62^	2019	Region‐CNN (R‐CNN)	CBCT	10 lung cancer patients (3500 raw CBCT projection images)	The performance of the model was lower at lateral angles when larger amounts of fatty tissue obstructed the view of the diaphragm. The model could estimate the diaphragm apex positions with a mean error of 4.4 mm.
Jiang et al.^63^	2019	A non‐linear autoregressive model with exogenous input (NARX)	Gating system	7 lung cancer patients	Mean ± standard deviation was 82.32 ± 17.93%, 80.52 ± 18.00%, and 79.77 ± 18.42% of three different prediction horizons, 600 ms, 800 ms, and 1 s, respectively.
Lin et al.^24^	2019	A model was a combination of four base machine learning algorithms such as the RF, MLP, LightGBM, and XGBoost.	4DCT and the Electronic Health Record	150 lung cancer patients	The maximum MAE and RMSE were in the superior‐inferior (SI) direction with 1.23 and 1.70 mm, respectively.
Hirai et al.^64^	2019	DNN (CNN)	4D‐DRR (digitally reconstructed radiography) images derived from 4DCT	5 lung cancer patients and 5 liver cancer patients. Each 4DCT contains 10 respiratory phases	Averaged track accuracy was 1.64 ± 0.73 mm Accuracy for liver cases was 1.37 ± 0.81 mm and for lung cases was 1.9 ± 0.65 mm Computation time was less than 40 ms
Lei et al.^7^	2020	The transNet model consists of three modules (encoding, transformation, and decoding modules)‐ GAN	3D CT generated from 2D CT	24 lung cancer patients	The mean value of the center of mass distance between manual tumor contours on the ground images and corresponding 3D CT images derived from 2D projection was 1.26 mm, with a maximum deviation of 2.6 mm. The peak signal‐to‐noise ratio was 15.4 ± 2.5 decibels (dB) and the structural similarity index metric within the tumor region of interest was 0.839 ± 0.090.
Wei et al.^65^	2020	CNN	A 3D moving mask derived from 3DCT was projected onto the 2D images	15 lung cancer patients	The average tumor localization error was less than 1.8 and 1.0 mm in SI and LR directions, respectively.
Mori et al.^66^	2020	DNN	4DCT derived from 3DCT	Train set: 2420 thoracic 4DCT from 436 patients Test set: 20 lung cancer patients	The averaged tracking positional errors were 0.56 , 0.65 , and 0.96 mm in the X, Y, and Z directions, respectively.
Wang et al.^67^	2020	Convolutional‐RNN	kV projections of CBCT	13 lung cancer patients	The computation time was 20 ms using a high‐performance computer. The 3D localization error was 1.3 ± 1.4 mm.
Sakata et al.^68^	2020	Extremely randomized trees (ERT)	4DCT	8 lung cancer patients	The average tracking positional accuracy was 1.03 ± 0.34 mm (mean ± standard deviation, Euclidean distance) and 1.76 ± 0.71 mm (95th percentile).
Dai et al.^69^	2021	Markov‐like network	US	The Cardiac Acquisitions for Multi‐structure Ultrasound Segmentation (CAMUS) dataset includes 2D US images from 450 patients. The Challenge on Liver Ultrasound Tracking (CLUST) dataset consists of 63 2D and 22 3D image sequences from 42 patients and 18 patients	CLUST dataset: A mean tracking error of 0.70 ± 0.38 mm for the 2D point landmark tracking and 1.71 ± 0.84 mm for the 3D point landmark tracking. CAMUS dataset: A mean TE of 0.54 ± 1.24 mm for the landmarks in the left atrium
He et al.^70^	2021	ResNet generative adversarial network (GAN) was used to learn the mapping between 2D kV and DRRs. Motion tracking was performed via registration to the reference spine DRR.	kV x‐ray from CBCT	Train set: 20 patients (1347 2D kV thoracic and lumbar region). Test set: 4 patients (226 2D kV images)	The model was able to enhance image quality for submillimeter accuracy. The decomposed spine image was matched with the ground truth with an average error of 0.13, 0.12, and a maximum of 0.58, and 0.49 in the x‐ and y‐directions respectively.
Momin et al.^30^	2021	R‐CNN, VoxelMorph, U‐Net, networks without global and local networks, and networks without attention gate strategy	4DCT	First experiment (Training set: 20 4DCT, Test set: additional 20 4DCT) The second experiment (training set: 40 4DCT, test set: 9 additional unseen 4DCT). Each 4DCT contains 10 breathing phases	The method was evaluated against several other networks, including VoxelMorph, U‐Net, and networks without global or local networks or attention‐gate strategies. The Dice similarity coefficients of experiments 1 and 2 were higher than those achieved by VoxelMorph, U‐Net, networks without global and local networks, and networks without attention gate strategy. Specifically, experiment 1 achieved a coefficient of 0.86 compared to 0.82, 0.75, 0.81, and 0.81 achieved by the aforementioned methods, and experiment 2 achieved a coefficient of 0.90 compared to 0.87, 0.83, 0.89, and 0.89 achieved by the aforementioned methods.
Pohl et al.^29^	2021	RNN, linear predictor (LP), least mean squares (LMS)	4D CBCT and 4DCT	4 lung cancer patients (Chest 3D 16‐bit image sequences). Each sequence had 10 3D images of the chest at different phases of breathing	RNN was superior to LP and LMS. The maximum prediction error for RNN, LP, and LMS was 1.51 , 1.80 , and 1.59 mm respectively. RMSE was 0.444 , 0.449 , and 0.490 mm for RNN, LP, and LMS, respectively. The Jitter of RNN, LP, and LMS was 2.59 , 2.59 , and 2.63 mm, respectively.
Terpstra et al.^26^	2021	A multiresolution CNN called TEMPEST	MRI	27 lung cancer patients (training set:17, validation set:5, test set: 5). Also, the model was evaluated using the publicly available 4DCT dataset.	Compared to the self‐navigation signal using 50 spokes per dynamic (366× undersampling), the model was able to provide more accurate motion estimation results. Deformation vector fields were estimated to be within 200 ms, including MRI acquisition. The target registration error of the model on 4DCT without retraining was 1.87 ± 1.65 mm.
Liu et al.^71^	2022	NuTracker model using MLP	4DCT	7 lung cancer patients with gold fiducial markers	The proposed model had 26% and 32% improvement over the predominant linear methods with the mean localization error of 0.66 mm and < 1 mm at the 95^th^ percentile.
Lombard et al.^72^	2022	LSTM, LR	MRI	Training and validation set: 70 patients from group 1. The test set includes 18 patients from Group 1 and 3 patients from Group 2	LSTM outperformed compared to the LR model. For the 500 ms forecasted interval, a mean RMSE of 1.20 and 1.00 mm were obtained for LSTM, while the LR model yielded a mean RMSE of 1.42 and 1.22 mm for the group 1 and group 2 testing sets, respectively.
Zhang et al.^21^	2022	Light gradient boosting machine‐based recursive feature elimination with radiomics features	4DCT	67 lung cancer patients	Mean ± standard deviation was 0.8 ± 0.126, 0.829 ± 0.14, and 0.864 ± 0.086 for thresholds of 0.7, 0.8, and 0.9, respectively. The specificities were 0.771 ± 0.114, 0.936 ± 0.0581, and 0.839 ± 0.101. The area under the curve (AUC) was 0.837, 0.946, and 0.877, respectively.
Hindley et al.^73^	2023	Voxelmap network	CBCT	2 lung cancer patients (6120 images for training, 680 images for validation, and 680 images for testing).	The mean errors of 3D tumor motion prediction were 0.1 ± 0.5, −0.6 ± 0.8, and 0.0 ± 0.2 mm in the left‐right, SI, and anterior‐posterior directions respectively.
Huttinga et al.^74^	2023	Gaussian process	MRI (MR‐linac)	One phantom, one healthy volunteer, one patient	The root‐mean‐square distances and mean end‐point‐distance with the reference tracking method were less than 0.8 mm for all cases.
Lombard et al.^75^	2023	Classical LSTM network (LSTM‐shift), Convolutional LSTM (convLSTM), and convLSTM with spatial transformer layers (convLSTM‐STL)	MRI	88 patients (training:52, validation: 18, and test:21)	The LSTM‐shift model was found to be significantly better than other models. The maximum RMSE of LSTM‐shift was 1.3 ± 0.6 mm. convLSTM and convLSTM‐STL yielded the maximum RMSE of 1.9 ± 1.1 and 1.9 ± 1.0 mm, respectively.
Zhou et al.^76^	2023	DNN (CNN)	kV x‐ray (2D‐DRR images)	10 lung cancer patients (2250 DRRs)	The mean calculation time was 85 ms per image. The median value for the 3D deviation was 2.27 mm overall. There is a 93.6% chance that the 3D deviation is less than 5 mm.
Li et al.^20^	2023	multilayer perceptron (MLP), wide and deep (W&D), categorical boosting (Cat), light gradient boosting machine (Light), extreme gradient boosting (XGB), adaptive boosting (Ada), random forest (RF), decision tree (DT), logistic regression via stochastic gradient descent (SGD), gaussian naive bayes (GNB), support vector classifiers (SVC), linear support vector classifiers (linearSVC), and K‐nearest neighbor (KNN)	CT	108 lung cancer patients	The best result in terms of AUC was obtained by SVC (0.941). Linear SVC provided the best outcome in terms of sensitivity (0.848). The best specificity results were achieved using MLP (0.936). In general, MLP demonstrated the best classification performance and stability among all models.
Liang et al.^77^	2023	RNN	Photonic delay‐line reservoir computer	76 patients	With a 333 ms look‐ahead time, normalized mean square error (NMSE) = 0.025, MAE = 0.34 mm, RMSE = 0.45 mm, an average therapeutic beam efficiency (TBE) of 94.14% for an absolute error (AE) < 1 mm, and 99.89% for AE < 3 mm.
Zhang et al.^78^	2023	An encoder‐decoder deep learning model	4DCT	10‐phase 4DCT of 58 patients + six‐phase 4DCT of five patients	Lung contour deformation features (LCDFs) were able to predict the internal targets with a localization error of 2.6 ± 1.0 mm. A localization error of 4.7 ± 0.9 mm and real‐time performance of 256.9 ± 6.0 ms was achieved by the cascade ensemble model (CEM).
Dai et al.^79^	2024	GAN was used for data augmentations. To predict the target position, CNN was applied.	2D‐DRRs / real medical 2D projections	Patients from TCIA, and CIRS phantom data	The computation time of the proposed model was < 51 ms. For patient evaluation, the maximum localization error was 0.72 mm in the AP direction. For the phantom study, the localization error was less than 0.10 mm.
Fu et al.^80^	2024	Conditional Generative Adversarial Network (cGAN), known as Pix2Pix. To generate high‐quality images. Then template matching was used for tumor tracking	4DCT and CBCT were used to build and verify patient‐specific models respectively.	9 lung cancer patients	The average tracking error was 0.8 ± 0.7 mm in the SI direction and 0.9 ± 0.8 mm in the in‐plane left‐right (IPLR) direction.

### 3.2 Abdomen

Radiation therapy for gastrointestinal cancers often faces two main physical challenges. The first is the proximity of the tumor(s) to many essential OARs such as the duodenum, stomach, small intestine, kidneys, or spinal cord at the level of the abdomen. The second is the mobility typical of both the target and nearby OARs.^81^ In the abdomen, respiration, peristalsis, and variable organ filling result in variation in target position and organ deformation. The advent of advanced treatment techniques such as volumetric modulated arc therapy (VMAT) and SBRT solved the first challenge by providing highly conformal 3D dose distributions. However, the effective delivery of such a conformal dose to the target requires careful motion management.^32^, ^81^ In addition to gating respiratory and breath hold, using an abdominal compression plate is a primary strategy for motion management in the abdomen. However, upper lobe lesions are subject to motion in non‐diaphragmatic breathers. To resolve these issues, several AI methods have been suggested to augment motion‐tracking techniques due to their capability to assess several aspects simultaneously. The AI‐based approaches in the abdominal region are summarized in Table 2. This table provides information on the dataset, including the number of patients, publication year, AI model, input, image modality, and key outcomes, similar to Table 1. As shown in Table 2, different image modalities especially MRI are used in the abdominal region. Both CT and MRI are capable of identifying fatty lesions and their proximity to surrounding structures. However, some soft tissue tumors could not be delineated from normal muscle with CT. On the other hand, MRI is a particularly useful image modality in this context because it is highly effective in imaging and evaluation of various soft tissue, which is abundant in the abdominal regions.^82^ US can also be used for monitoring anatomical movements in soft tissue and is particularly effective in monitoring hepatic and pancreatic targets due to site accessibility and the absence of osseous obstruction.^18^

**TABLE 2:** Summary of publication using artificial intelligence for real‐time abdomen tumor tracking.

Author	Year	Algorithm	Image modality	No. of patients	Key findings in results
Gou et al.^83^	2016	Dictionary learning model	MRI	3 pancreatic patients and two healthy volunteers (total of 12 imaging volumes)	The dictionary method improved the auto‐segmentation, at least 1 of the auto‐segmentation methods with Dice's index > 0.83 and shift of the center of the organ was less than equal to 2 mm.
Stemkens et al.^84^	2016	PCA	MRI	Phantom and 7 healthy volunteers (Pancreas and kidney)	An average error of 1.45 mm with a temporal resolution < 500 ms was achieved.
Dick et al.^85^	2018	ANN with Leave‐one‐out‐cross‐validation	4DCT	4D extended cardiac‐torso (XCAT) phantom and 8 liver patients	The averaged RMSE was 1.05 ± 1.14 and 2.26 ± 2.4 mm for phantom and patients’ data respectively.
Huang et al.^86^	2019	k‐dimensional‐tree‐based nearest neighbor search	US	57 different anatomical features were acquired from 27 sets of 2D ultrasound sequences.	The mean tracking error between manually annotated landmarks and the location extracted from the indexed training frame is 1.80 ± 1.42 mm. Adding a fast template matching can reduce the mean tracking error to 1.14 ± 1.16 mm.
Huang et al.^87^	2019	FCN with convolutional LSTM (CLSTM)	US	The train set was 25 and the test set was 39 liver cancer patients	The mean and maximum tracking error were 0.97 ± 0.52 and 1.94 mm, respectively. The tracking speed using GPU was from 66 to 101 frames per second.
Zhao et al.^88^	2019	A patient‐specific region‐based convolutional neural network (PRCNN)	2D‐DRRs	2 pancreatic cancer patients (2400 DRR datasets)	The mean absolute difference between the model‐predicted and the actual positions < 2.60 mm in all directions. Lin's concordance correlation coefficients between the predicted and actual positions were > 93%.
Liang et al.^89^	2020	FCN (U‐Net)	Cyberknife Synchrony (real x‐ray images)	13 liver cancer patients (5927 images)	The mean centroid error between the predicted and the ground truth was 0.25 ± 0.47 pixels on the test dataset. The maximum mean translation was seen in the SI direction with 13.1 ± 2.2 mm.
Liu et al.^90^	2020	SVM	Cyberknife® Synchrony	148 liver cases and 48 cases of other anatomical sites (e.g., Kidney, pancreas)	The sensitivity, precision, specificity, F1 score, and accuracy are 0.81 ± 0.09, 0.85 ± 0.08, 0.80 ± 0.11, 0.83 ± 0.06, and 0.80 ± 0.07, respectively. An AUC of 0.87 ± 0.05 was achieved.
Roggen et al.^91^	2020	Mask R‐CNN	2D kV derived from CBCT	Train set: 12 abdominal cancer patients (903 images). Test set: 1 patient (49 images) and one phantom with vertebrae	This study presents a fast DL model for detecting landmarks in vertebrae and evaluates its accuracy in detecting 2D motion using projection images taken during treatment. The proposed network was able to detect the motion in translational shifts (within a range of 1.5 mm) and rotational variations > 1 degree.
Terpstra et al.^92^	2020	The convolutional neural network called SPyNET	MRI	7 abdomen, 40 liver, 62 kidney, and 26 pancreas cancer patients (200 images)	Combining non‐uniform fast Fourier transform with SPyNET resulted in acceptable performance for 25‐fold accelerated data, yielding an imaging frame rate of 25 Hz while keeping the RMSE within 1 mm.
Bharadwaj et al.^31^	2021	Upgraded Siamese neural network using Linear Kalman filter (LKF)	US	CLUS	The proposed model enhanced the Siamese neural network by resolving the constant position model issue and improving robustness. To improve the original architecture, LKF was added to include the missing motion model.
Romaguera et al.^93^	2021	Encoder‐decoder network	MRI, US	MRI: 25 volunteers, and 11 patients diagnosed with hepatocellular carcinoma. US: 20 volunteers.	The model used image surrogates for volumetric prediction and yielded mean errors of 1.67 ± 1.68 and 2.17 ± 0.82 mm for unseen MRI and US patient datasets, respectively.
Shao et al.^94^	2021	U‐Net	3D‐DVFs estimated by the 2D‐3D deformable registration	34 liver cancer patients (train set:24, test set:10)	The model obtained the mean center of mass error of 4.7 ± 1.9 , 2.9 ± 1.0 , and 1.7 ± 0.4 mm, the average DICE coefficients of 0.60 ± 0.12, 0.71 ± 0.07, and 0.78 ± 0.03, and the mean Hausdorff distances of 7.0 ± 2.6 , 5.4 ± 1.5 , and 4.5 ± 1.3 mm, for 2D‐3D, 2D‐3D deformable registration with biomechanical modeling, and DL model prediction with biomechanical modeling techniques, respectively.
Wang et al.^33^	2021	LSTM, SVM	4D US and template matching to track the motion	7 volunteers	LSTM was superior to SVM with RMSE less than 0.5 mm at a latency of 450 ms for the prediction of respiratory motion and internal liver motion of < 0.6 mm.
Liu et al.^95^	2022	DNN	MRI	7 liver cancer patients	The proposed model increased the image quality in terms of structural similarity index, peak signal noise ratio, and mean square error. The median distance between the predicted model and the ground truth in the SI direction was 0.4 ± 0.3 and 0.5 ± 0.4 mm for cine and radial acquisitions, respectively.
Shao et al.^96^	2022	RegNet, KS‐RegNet‐nup, and KS‐RegNet	MRI	Eight cases from an open‐access multi‐coil k‐space dataset (OCMR) were used for the cardiac dataset, 9 liver cancer patients for the abdominal dataset were selected.	KS‐RegNet was found to have better, and more stable performance compared to other models.
Shao et al.^97^	2022	Graph convolutional network or GCN	2D images derived from 3DCT	10 liver cancer patients and each patient had 10 respiratory phases	The mean localization error was less than 1.2 mm. This indicates the potential of the model for tumor tracking.
Ahmed et al.^32^	2023	CNN, YOLO, and hybrid CNN‐YOLO	Sub‐images of kV x‐ray	13 patients (training data (44 fractions, 2017 frames). Test data (42 fractions, 2517 frames)).	The MAE and RMSE of all 3 models were less than 0.88 ± 0.11 and 1.09 ± 0.12 mm, respectively.
Dai et al.^98^	2023	CNN	Optical surface monitoring, and kV x‐ray	7 liver cancer patients	The maximum MAE and RMSE were observed in the SI direction (3.12 ± 0.80 and 3.82 ± 0.98 mm, respectively).
Hunt et al.^99^	2023	DL (VoxelMorph and U‐Net), Affine, b‐Spline, and demons	MRI	21 patients with abdominal or thoracic tumors (> 629 000 frames from 86 treatment fractions)	The DL model provided better results compared to conventional methods. The RMSE was 0.067, 0.040, 0.036, and 0.032 for affine, b‐spline, demons, and DL respectively.
Shao et al.^100^	2023	DL‐based framework (Surf‐X‐Bio)	kV x‐ray and surface imaging	34 liver cancer patients	The Surf‐X‐Bio can precisely monitor liver tumors through a combination of surface and x‐ray imaging compared to surface‐image‐only and x‐ray‐only models.
Weng et al.^101^	2023	Convolutional long short‐term memory (convLSTM)	2D cine magnetic resonance (cine‐MR) imaging	17 cine‐MRI datasets including stomach, liver, pancreas, and kidney	The predicted images achieved average structural similarity index (SSIM) values of 0.54, 0.64, 0.77, and 0.66 for the stomach, liver, kidney, and pancreas, respectively. The computation time to generate a predicted image for smaller ROIs, such as the stomach, is less than 10 ms. For larger ROIs, such as the liver, the computation time is around 100 ms using a single GPU.
Xiao et al.^102^	2023	Downsampling‐Invariant Deformable Registration (D2R)	MRI	43 liver cancer patients for building model and 5 patients for validation	The average ROI tracking error was highest in the SI direction, at 1.18 ± 1.20 mm. The whole construction process was less than 500 ms, and the simulated image acquisition frequency was greater than 3 Hz.

### 3.3 Pelvis

According to the American Cancer Society, prostate cancer is the most common malignancy of men in the US, accounting for 27% of the total diagnoses of all sites.^36^ Thus, prostate cancer has been widely studied. Radiotherapy is indicated as a primary and salvage treatment for prostate cancer in both early and advanced diseases. The major challenge of prostate radiation therapy is unpredictable intrafraction prostate motion due to variable rectal and bladder filling. Target position uncertainties in prostate cancer radiotherapy are usually addressed by assigning a margin around the target volume.^103^, ^104^, ^105^ However, without continuous monitoring and intervention, intrafraction motion can cause a geographic miss in approximately 10% of SBRT prostate therapy cases.^5^ Various strategies have been proposed for intrafraction motion monitoring such as US, kV/kV imaging, infrared cameras, implanted fiducial markers with in‐room imaging, CBCT, and MRI.^104^, ^105^ Among these, fiducial markers used in conjunction with x‐ray imaging are most commonly employed, allowing for real‐time target localization and tracking.^106^, ^107^ However, even with appropriate use, marker migration within and outside of the prostate can reduce the dosimetric coverage of the target volume and increase the dose to OARs.^107^ Several authors have shown that AI‐based models can be a potential tool to resolve complications associated with marker‐based techniques. The summary of AI approaches to motion management in the pelvic region is presented in Table 3. This table presents an overview of the research methodologies, data sets used, and key findings of each study.

**TABLE 3:** Summary of publication using artificial intelligence for real‐time pelvic tumor tracking.

Author	Year	Algorithm	Image modality	No. of patients	Key findings in results
Zhao et al.^108^	2019	DNN	CT	10 prostate cancer patients	Differences between the positions, predicted by DNN and ground truth positions are (mean ± standard deviation) 1.58 ± 0.43 , 1.64 ± 0.43 , and 1.67 ± 0.36 mm in anterior‐posterior, lateral, and oblique directions, respectively.
Zhu et al.^109^	2019	Deep convolutional neural network	US	83 image pairs from 5 prostate cancer patients	CNN registration errors were < 5 mm in 81% of the cases. In contrast, manual registration errors were less than 5 mm in 61% of the cases. Also, advanced normalized correlation registration errors were < 5 mm only in 25% of the cases.
Mylonas et al.^110^	2019	CNN(AlexNet)	kV x‐ray	Training set (a phantom and 5 intrafraction images from three prostate cancer patients). Test set (12 fluoroscopic intrafraction images from 10 prostate cancer patients).	The method was effective for continuous fluoroscopic imaging where the markers were in the tracking window in subsequent image acquisition.
Amarsee et al.^111^	2021	You Only Look Once (YOLO) convolutional neural network	kV x‐ray	One phantom	The fiducial marker seeds were successfully detected in 98% of images from all gantry angles; the variation in the position of the seed center was within ± 1 mm. The percentage difference between the ground truth and the detected seeds was within 3%.
Fransson et al.^103^	2021	Principal component analysis (PCA) (unsupervised machine learning)	MRI (MR‐Linac)	9 healthy male volunteers	The cumulative variance of the eigenvalues from the PCA showed that 50% or more of the motion is explained in the first component for all subjects.
Nguyen et al.^112^	2021	Kalman Filter framework	kV x‐ray	17 prostate cancer patients (536 trajectories)	The maximum RMSE (without noise) was obtained at 0.4 ± 0.1 mm. With noise, the RMSE was 1.1 ± 0.1 mm.
Motley et al.^107^	2022	CNN (You Only Look Once (YOLO))	2D projections from CBCT images	14 prostate cancer patients (∼ 20 000 pelvis kV projection images)	The detection efficiency of the model was 96% with an RMSE of 0.3 pixels.
Chrystall et al.^106^	2023	CNN	MV‐based IGRT	29 prostate cancer patients	The AUC of 0.99, the sensitivity of 98.31%, and the specificity of 99.87% were achieved. The mean absolute geometric tracking error was 0.30 ± 0.27 for lateral and 0.35 ± 0.31 mm for SI directions of the MV images.

The objective of Tables 1, 2, 3 is to present a comprehensive view of the subject matter while offering a representative sample of publications, rather than an exhaustive list. As a result, studies that cover multiple treatment sites or provide similar information are only included once in the Tables.

## 4 ARTIFICIAL INTELLIGENCE FOR MOTION TRACKING IN RADIOTHERAPY

### 4.1 Classic machine learning based motion tracking algorithms

ML is a subfield of AI that utilizes algorithms to analyze input data and learn from them to provide recommendations and decisions. The typical workflow of classic ML includes data collection, data pre‐processing, dataset (training, validation, and test) building, evaluation, and finally, deployment to production. In radiotherapy, classic ML methods can assist in analyzing different aspects of target motion to predict future positions and optimize treatment delivery. According to the statistics of this review (Figure 3) in this context, NN, SVM, and boosting algorithms are the most popular classic ML algorithms for tumor tracking. NN is of further interest due to its ability to capture both dynamic and structural phenomena, noise suppression, edge detection, and image enhancements.^43^, ^57^, ^113^ Moreover, in neural network environments, a neuron unit or cell is considered directionally selective. It responds, solely when motion occurs in a particular direction while remaining unresponsive, or silent, to motion in all other directions.^114^ These features make it perhaps the best choice among other classic ML techniques for finding useful solutions that require less human intervention. The Support Vector Machine, or SVM, is widely used due to its exceptional performance, flexibility, and efficiency—even when working with limited datasets. It has several parameters that are essential to finding the most optimal model, including ε, which denotes how much error is allowed per training data, C, which balances the amount of penalty imposed on margin violations, and the kernel coefficient, or γ.^115^ The SVM's ability to handle nonlinear classification in a high‐dimensional (kernel) space makes it particularly useful for processing nonlinear problems and real‐time dynamic predictions, which greatly improves its overall performance.^23^, ^33^, ^116^ In addition to NN and SVM, boosting^21^ models are also being successfully applied to perform motion predictions. Boosting model increases the weights of incorrectly classified objects, allowing a weak classifier to focus on their detection. The user can set the frame in which the tracking object is located and the classifier scores the surrounding detection pixels to determine the new object's position.^117^

The descriptions and drawbacks of common classic ML approaches in the context are summarized in Table 4.

**TABLE 4:** A list of more common classic machine learning algorithms used for tumor tracking position.

Models	Description	General drawbacks
Neural network (NN)	A typical neural network has three layers including an input layer, a hidden layer, and an output layer. This system can process information and adjust to changing situations to optimize its performance in real time. It yields great accuracy for nonlinear and irregular patterns. It can be used for regression and classification. ANN includes MLP, QNU, Adaptive neuro‐fuzzy inference system (ANFIS), Non‐linear autoregressive with exogenous, wide, and deep (W&D)	Tend to overfit on small‐size datasets Memorize the training data and do not generalize well the learned knowledge to new or different data^117^, ^118^ Simple ANN is lack of spatial context (which is a vital draw back for task like image ;lsegmentation)
Support vector machine (SVM)	The SVM is a supervised ML algorithm that performs classification and regression by finding the best line or decision boundaries to separate data into classes. The algorithm is a kernel‐based model to solve linear and non‐linear problems.^115^	Strongly depends on the kernel Sensitive to noise Not suitable for large dataset
Boosting	Boosting, known as a shallow predictor, is an ensemble modeling technique that combines a set of weak classifiers to build a strong classifier. This helps to reduce training errors, and biases plus improve accuracy. It includes Adaboost, XGBoost, Gradient tree boosting	‐Sensitive to outliers ‐Almost impossible to scale it up ‐Low speed

#### 4.1.1 Related works using classic machine learning

Neural network approaches have been used in several studies to predict target position during treatment delivery. Isaksson et al.,^41^ for example, use the positions of external surrogate markers as input for adaptive neural networks. The study demonstrated that the adaptive neural network provides a more accurate estimation of tumor position compared to fixed and adaptive linear filters. A subsequent study conducted by Murphy and Dieterich^43^ confirmed the advantage of an adaptive neural network by comparing it against the linear adaptive filter. Krauss et al.^51^ performed a study on 12 samples of breathing data using linear regression (LR), kernel density estimation, SVM, and ANN. They indicated there were small differences between the models (Table 1). To improve the accuracy of ANN, some authors used an adaptive neuro‐fuzzy inference system (ANFIS), which combines the benefit of both neural network and fuzzy logic systems.^42^, ^49^, ^58^ Kakar et al.^42^ used ANFIS to predict respiratory motion both more precisely and more quickly. Yan et al.^44^ developed a technique using ANN to predict tumor position by correlating the internal target position and an external surrogate. The proposed technique assumes a consistent correlation between internal and external movements, allowing for their prediction errors to be correlated with a linear model. In 2008, Cui et al.^23^ proposed that an SVM is a potentially accurate and efficient algorithm for predicting target position. Riaz et al.^48^ analyzed the performance of multi‐dimensional adaptive filters versus a support vector machine (SVM) to predict lung tumor motion. They showed the superior performance of the SVM model with RMSE < 2 mm at 1‐s latency. Lin et al.^47^ further demonstrated the superiority of an ANN approach over SVM (Table 1). An extremely randomized tree (ERT) algorithm can also be used to predict tumor motion. Sakata et al.^67^ trained an ERT for position prediction of lung tumors using digitally reconstructed radiography (DRR) as inputs. They also used sliding window classification to provide a tumor likelihood map. The model was tested on 4DCT of eight patients and yielded an accuracy of 1.0 ± 0.3 mm (Table 1). In 2015, Bukovsky et al.^57^ presented a combination of quadratic neural unit (QNU) with modification of the Levenberg‐Marquardt (L‐M) algorithm to improve the accuracy of prediction. The authors achieved a prediction error of less than 1 mm on average of internal tumor position in total treatment time. Moreover, they indicated that QNU with the Levenberg‐Marquardt (L‐M) algorithm is faster and can yield more accurate results than multilayer perceptron (MLP). In contrast, a study conducted by Li et al.^20^ showed that the MLP model had better classification performance and stability for both lung and liver tumors compared to other models. The authors compared thirteen algorithms such as MLP, wide and deep (W&D), categorical boosting, light gradient boosting machine, extreme gradient boosting, adaptive boosting, random forest, decision tree, logistic regression via stochastic gradient descent, Gaussian Naive Bayes, SVM, linear support vector classifiers, and K‐nearest neighbor. All models were developed based on radiomic features extracted from CT images of 108 patients with lung cancer and 71 patients with liver cancer. Stemkens et al.^84^ proposed a patient‐specific method using a 3D motion model and fast 2D cine‐MR imaging to estimate abdominal motion. The motion model was obtained by performing a principal component analysis (PCA) on inter‐volume deformation vector fields (DVFs) that were derived from a pre‐treatment 4D MRI scan.

### 4.2 Deep learning based motion tracking architecture

**FIGURE 4:**
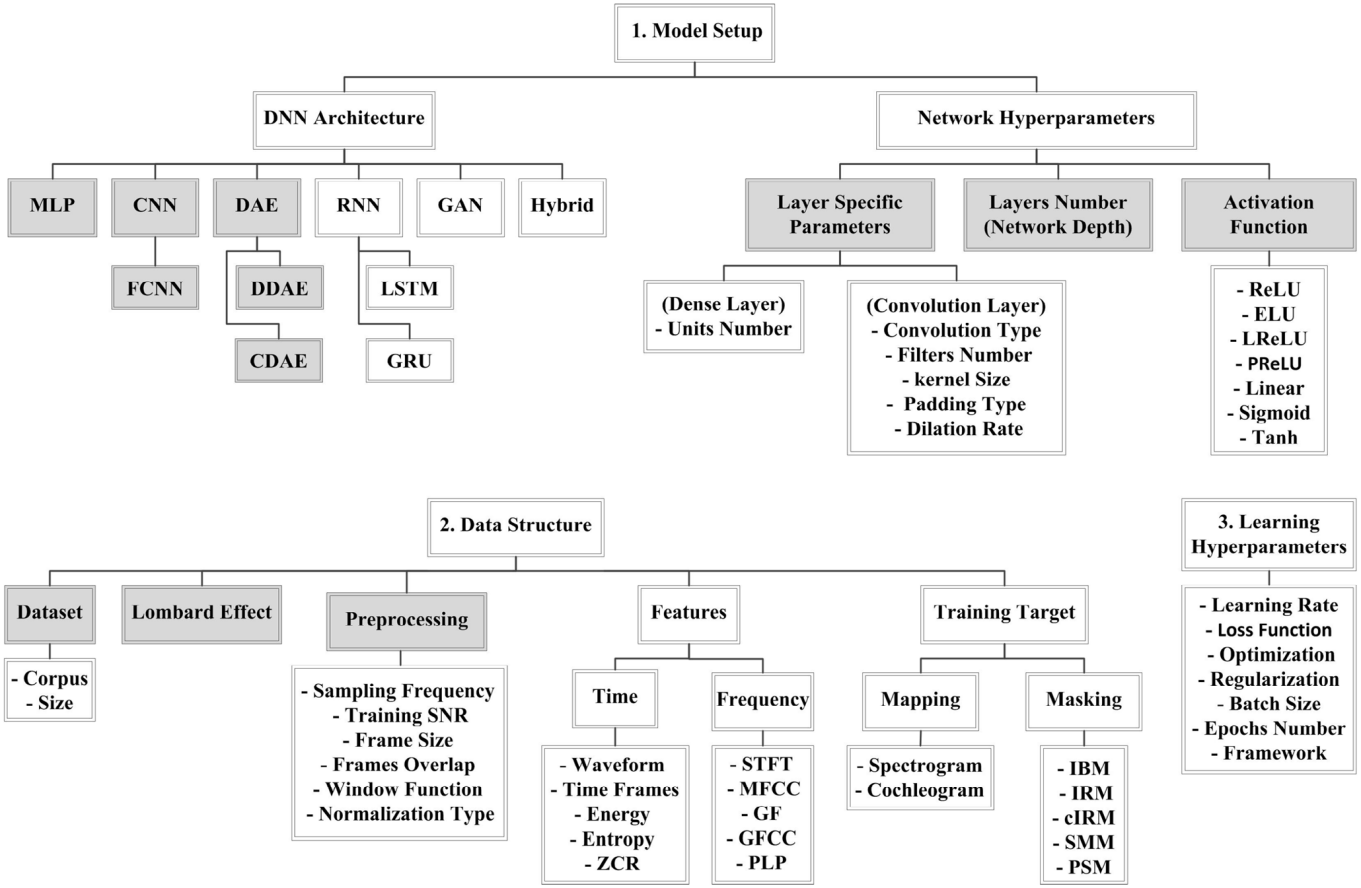
The three main factors affecting deep learning performance are model setup, data structure, and learning hyperparameters.^121^

DL, a subclass of ML, uses a stack of processing layers with non‐linear units that extract higher‐level features from inputs. The advantage of DL over classic ML is that DL uses artificial neural networks to automatically generate features from data that are suitable for the task. The multilayered structure of DL enables it to self‐train based on inputs and desired outputs.^120^ The workflow of DL is similar to classic ML and starts with data acquisition and preprocessing followed by building and training the model, optimization, evaluation, and predictions or inference. Figure 4 illustrates the three main factors that can impact the performance of deep learning models. These factors can be categorized into the model setup, data structure, and learning hyperparameters.^121^

Our systematic review findings have confirmed that after careful analysis, we could not find any two studies that identified the same features or used the same database, even if they used the same image modality and cancer type. The performance of DL studies is influenced by hardware specifications and imaging protocols, in addition to variability between individual patients such as anatomic geometry, and tumor location.^122^, ^123^, ^124^ These factors make it challenging to determine which DL model is superior to the others. However, our statistical analysis (Figure 3) demonstrated that CNNs, U‐Net, and GANs have been extensively applied in motion management. These three groups are not entirely different from each other but represent stepwise increases in architectural complexity.^125^ A typical CNN has an initial input layer, and a final output layer with several intervening “hidden” layers connecting the input and output. In CNNs, the hidden layers extract higher‐level image “features” from the input image, typically across several resolutions, capturing detail at several spatial scales. In other words, CNN is typically composed of three types of layers: convolution, pooling, and fully connected layers. The convolution and pooling layers perform feature extraction, while a fully connected layer, maps the extracted features into the final output, such as classification.^109^, ^126^ Convolutional neural networks, or CNNs, were mainly designed to efficiently handle data that has a grid‐like structure, such as images. Due to their exceptional ability to recognize objects, CNNs have become a popular choice for various computer vision tasks, including image recognition and object detection.^101^, ^127^ Notably, CNNs have been applied in medical image analysis to predict the position of tumors (Tables 1, 2, 3). CNN is largely used when the whole image is needed to be classified as a class label. However, biomedical image analysis for target tracking requires the classification of each pixel. To solve this issue, Ronneberger et al. proposed a new architecture named U‐net in 2015.^28^ U‐Net, also called a fully convolutional network, is a CNN based on an autoencoder architecture and was designed for semantic segmentation in medical images, making it extremely good at removing specific information from images.^126^ CNN typically converts images into vectors for classification, but in U‐Net, an image is first compressed into a vector through a down‐sampling process, and then an up‐sampling procedure gradually reconstructs the vector back to the original image shape. This preserves the original structure and reduces distortion. U‐Net goes beyond simple pixel discrimination by projecting learned features onto the pixel space. However, U‐Net is unable to perform image classification. It is optimized for semantic segmentation tasks where each pixel of an image is classified instead of the image as a whole. Therefore, it needs a pixel‐level ground truth mask.^125^ Another popular DL architecture in motion management is GAN. The concept of GAN was initially developed by Ian Goodfellow et al. in 2014.^128^ One of the key advantages of using GANs is their ability to generate high‐quality synthetic 3D images to enhance the visibility of tumors that closely mimic the actual position of cancerous tissues. It is worth noting that GANs are not applicable in tumor tracking, but they assist in generating high‐quality images for tracking tumors. In recent years, several researchers have implemented GAN‐based models to generate 3D images from 2D kV images^79^ or US images.^68^ These studies have demonstrated the ability of GANs to capture complex tumor motion patterns from training data and generate realistic 3D images simulating motion direction and calculating associated speed. This can be particularly valuable in real‐time tumor tracking, where a detailed and accurate representation of cancerous tissues is crucial to providing accurate treatment. GANs achieve this by training two neural networks simultaneously: a generator network and a discriminator network. The generator network uses random noise to generate synthetic images that are as close to reality as possible, while the discriminator network tries to determine whether the generated image is fake or real.^125^ A GAN is called adversarial because this setup forms a “min‐max” game, driving both networks to improve continuously until the discriminator cannot easily differentiate between real and generated data. GANs are particularly useful in generating high‐quality synthetic images under unsupervised learning, which is helpful when labeled data is not available. They can also be used for data augmentation to produce new data samples that resemble the training data distribution.^129^

The descriptions and downsides of common DL architectures are summarized in Table 5.

**TABLE 5:** A list of more common deep learning methods used for tumor tracking position.

Models	Description	General drawbacks
CNNs	CNNs are distinguished by their convolutional layers, which operate based on the prior assumption that neighboring pixels are correlated, effectively capturing spatial and temporal dependencies in image data. They have been frequently used for various computer vision applications, such as image classification, object recognition, and image segmentation. Also, they have helped in forming many different applications such as AlexNet, VGG, ResNet, Inception, and DenseNet.	Require a large amount of labeled data to achieve a good performance Tend to be overfitted Hyper‐parameter selection has a great influence on its performance
U‐Nets	U‐Net is a variation of CNN, which has a special U‐shaped encoder‐decoder network architecture with skip connections that directly connect encoder layers to their corresponding decoder layers. These connections help recover spatial information lost during down‐sampling in the encoder, which is vital for achieving accurate segmentation results. Moreover, U‐Net works well with a relatively small amount of medical imaging training data.	Mainly focuses on local features which may hinder its performance when a broader understanding of the entire image is necessary^128^
GANs	Generative Adversarial Networks (GANs) are a powerful class of neural networks that are applied for unsupervised learning. GAN is composed of a generator network and a discriminator. The discriminator is trained to give a high score for real images and a low score for synthetic images. Whereas the generator is trained to generate images that can fool the discriminator.	The training of GAN can be tricky due to the generator and discriminator being trained iteratively and balancing these two components is not quite easy^128^

#### 4.2.1 Related work using deep learning

**FIGURE 5:**
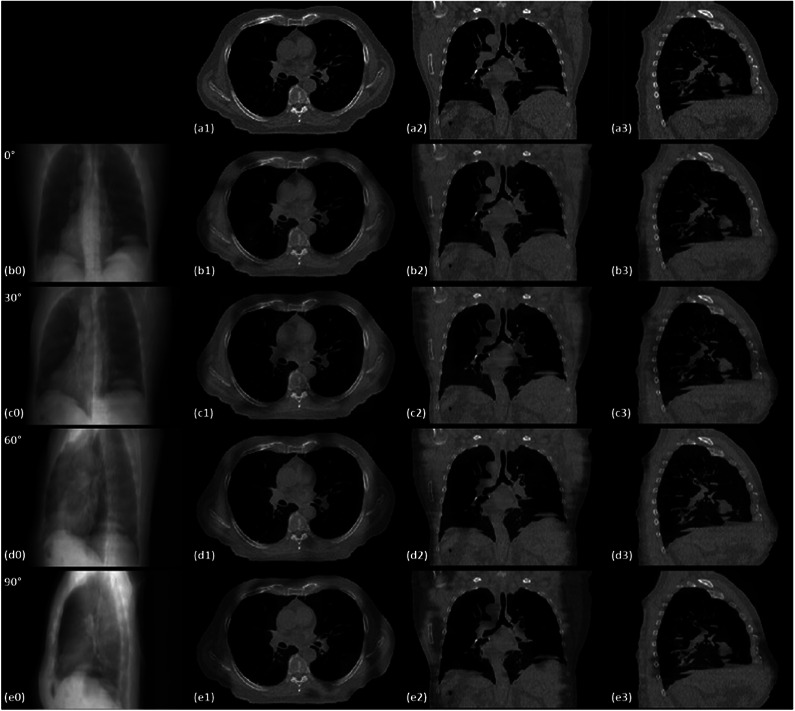
The 3D CT images are generated from a single 2D kV projection. (a1‐a3) are ground truth 3D CT in axial, coronal, and sagittal views. Rows (b‐d) demonstrate the projection data and corresponding generated 3D CT images for projection angles 0, 30, 60, and 90, respectively.^7^ (With permission).

Recent advancements in computer science and information technology have increased interest in DL‐based models for tracking target motion (Figure 1) so as to several algorithms have been trained for this task across different image modalities (Figure 2). In 2019, Zhao et al.^108^ conducted a study on 10 patients with prostate cancer who underwent either CBCT or orthogonal kV projections. The DL model used two networks: a region proposal network (RPN) and CNN. RPN was used to generate proposals for the region‐based CNN to reduce computation time and enable real‐time target detection. The two networks share all convolutional features. The study (Table 3) showed that highly accurate tumor localization can be achieved using CNN. However, Park et al.^58^ demonstrated that ANFIS was able to achieve an RMSE of 0.5 ± 0.8 mm using a 192.3 ms prediction, a 30.0% improvement over CNN. According to Park et al, the fuzzy logic component enhances the reasoning ability of the model when dealing with uncertainty.^58^ A study by Liang et al.^89^ used an automated framework to evaluate intrafraction motion in CyberKnife x‐ray images. The framework utilizes a fully convolutional network (FCN)‐based module to detect fiducial markers and perform semantic segmentation using a U‐Net architecture in full‐size x‐ray images. The 3D positions of the markers can then be reconstructed to evaluate intrafraction motion using a rigid transformation (Table 2). Wang et al.^33^ compared an LSTM approach against an SVM to estimate external respiratory motion and internal liver motion. The LSTM network was found to perform better on all planes. Edmunds et al.^61^ proposed to automatically segment the diaphragm using CBCT images for real‐time tracking of lung tumors. A Mask R‐CNN (Region Convolutional Neural Network) was trained on 3499 raw CBCT images from 10 patients with lung cancer. No manual intervention was required, and the model was able to track diaphragm motion in real‐time with a mean error of 4.4 mm. Several attempts were made to learn a joint mapping between partial views and 3D shapes. These efforts have established a path for connecting partial observations with high‐dimensional data within a comprehensively trained deep framework. In 2020, Lei et al.^7^ proposed a novel method named TransNet including encoding, transformation, and decoding modules to draw 3D CT images from 2D projections. Later, a fast volumetric imaging method with an orthogonal 2D kV/MV image pair using their modified deep learning method was developed for tumor localization in lung cancer radiation therapy.^130^ The result of this novel method (Table 1) suggests the feasibility and efficacy of the proposed technique to convert 2D to 3D images which can be a potential solution for real‐time lung tumor tracking in SBRT cases. The CT images were generated for one case by three different supervision strategies: (1) supervised by mean absolute error (MAE) and gradient difference (GD) losses (TransNet‐v1); (2) supervised by MAE, GD, and perceptual losses (TransNet‐v2); (3) supervised by a combination of MAE, GD, perceptual and adversarial losses (TransNet‐v3). Figure 5 shows the volumetric image generation based on different x‐ray projections acquired at different angles using TransNet‐v3.

**FIGURE 6:**
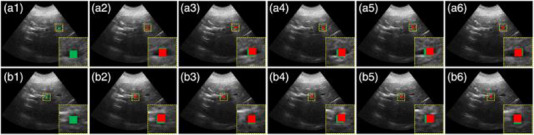
An illustrative example of 2D point‐landmark tracking. a1 and b1 are ground truth and the location of ground truth landmarks are shown in green boxes. (a2‐a5) and (b2‐b5) are predicted locations and red boxes indicate the predicted landmark positions in the two different landmark tracking.^68^ (With permission).

Liu et al.^70^ developed a new template network named NuTracker using an MLP comprising eight fully connected layers. The proposed model decomposes 4DCT images into template images and deformation fields using two coordinate‐based neural networks to generate predictions from spatial coordinates and surrogate states. Hirai et al.^63^ trained a deep neural network (DNN) to generate a target probability map (TPM) to predict the position of lung and liver tumors. Crops of the target and surrounding anatomy were produced from DRR images. These crops were used to produce the TPM. Accuracy was quantified using the Euclidian distance in 3D space between the calculated and reference tumor position (Table 1). US can provide real‐time volumetric images to track intra‐fraction motion during radiotherapy. Dai et al.^68^ applied a GAN‐based Markov‐like net for feature extractions from sequential US frames. These features are then used to estimate a set of DVFs via registration of both tracked and untracked frames. The positions of the landmarks in the untracked frames were determined by shifting landmarks in the tracked frame based on estimated DVFs (Figure 6). Zhang et al.^131^ developed a cascade deep learning model with an attention network, a mask region‐based convolutional neural network (mask R‐CNN), and a long short‐term memory (LSTM) network to track real‐time liver motion in US image‐guided radiation therapy (IGRT).

## 5 DISCUSSION

In the past, covering the target with large margins was required to compensate for intrafraction motion during radiotherapy, despite the risk of high‐dose delivery to OARs. In this context, several techniques have been developed for intrafraction motion management such as respiratory gating, fiducial implants, 4DCT, etc., to maintain target coverage while sparing OARs. In the following section, the limitations of current IGRT techniques and the use of AI models to perform real‐time volumetric imaging to improve clinical gain are discussed.

### 5.1 Image modalities and current limitations of IGRT techniques

Motion correction, before or during treatment, can be achieved with IGRT. The integration of IGRT with clinical linear accelerators has improved patient care and quality of life. X‐ray is the most common imaging modality employed in IGRT, primarily due to accessibility (Figure 2). X‐ray use began with the use of megavoltage beam (MV) portal imaging. However, due to the poor contrast resolution of MV images, in‐room kV imaging systems were introduced. In kV images, soft‐tissue contrast is higher, particularly when CBCT imaging is used. Planar kV (2D) and CBCT (3D) imaging are now typically available on modern standard‐equipped linear accelerators. Motion‐tracking systems equipped with IR‐based monitoring may be used in conjunction with x‐rays to further improve internal‐external correlation.^18^ For example, the CyberKnife Synchrony system utilizes stereoscopic x‐ray imaging to detect implanted clips and correlate them with external surrogates.^49^ Varian TrueBeam linear accelerators can also use respiratory gating and on‐board kV imaging to verify internal target anatomy at the start of the gated treatment window, guided by the RPM signal.^18^

Despite these advantages, limitations remain. X‐ray‐based kV and specifically MV imaging reliably capture osseous anatomy but fail to effectively resolve tumor motion within soft tissues.^132^ CBCT, which may be used to acquire a volumetric image of the patient's anatomy before treatment, cannot be used during treatment delivery. Regardless, CBCT is prone to severe artifacts, including streaking, beam hardening, and aliasing as well as those due to motion, which severely degrade image quality.^133^ While 4DCT represents a meaningful technological advance in the delivery of radiation therapy, it cannot provide information on variations between breathing cycles or variations occurring on a time scale beyond a magnitude of seconds.^134^ 4DCT/CBCT with implanted fiducial markers can aid real‐time tracking of tumor positions; however, this invasive method introduces additional risk associated with possible procedure site infection, fiducial marker migration, non‐trivial additional imaging dose exposure while increasing patient expense discomfort and inconvenience.^135^, ^136^ Furthermore, if fiducial markers are obscured by high‐density material like bone, surgical clips, or stents, their value is significantly diminished.^32^

MRI is the second most common imaging modality in clinical practice due to the integration of MR imaging with commercially available linear accelerators. The introduction of the MRI‐linear accelerator (MR‐linac) enables imaging both before and during treatment with greater soft tissue contrast relative to x‐rays, making it easier to differentiate targets from normal tissue.^103^ Notably, MR‐linac does not require the implantation of fiducial markers or the delivery of extra doses to the patient. However, its use is limited by the size of the imaging bore and is contraindicated in the presence of ferrous metal implants or cardiac pacemakers/defibrillators.

US imaging is another well‐established imaging technique that can provide real‐time volumetric images to track intra‐fraction motion without ionizing radiation.^86^ Yet, due to the poor penetration of US waves into deeper tissues, US cannot be used reliably in clinically important regions such as the skull and thorax.^137^

To accurately measure and analyze motion‐related data, two processes are always involved: determining the direction and calculating the magnitude of the velocity associated with that motion. While current IGRT techniques mostly provide information about the direction of the motion, they generally fail to cover the second aspect of the motion. These technologies usually use the target locations on 2D images to reconstruct the 3D position of the target.^88^ In this context, surrogates such as implanted fiducial markers, external infrared reflectors, or patients’ skin surface are used to track tumor position. However, these techniques either have unstable surrogate‐tumor correlation or are invasive. A study performed by Paganelli et al.^138^ to track tumor position in liver cancer patients showed that surrogate‐based tracking errors were between 7% and 23% (1.02–3.57 mm) and were significantly impacted by external motion parameters.^138^

With technological advancements, AI, especially deep learning, has made it possible to learn complex relationships and semantic features from data. First and foremost, AI technology has the capability to effectively capture a wide range of motion variables such as magnitude, amplitude, frequency, etc., and incorporate data from various sources to improve the efficiency and accurately track the tumor.^77^, ^139^ As an example, using AI in markerless image guidance is of great clinical relevance and would enable wide access to real‐time target tracking. A deep neural network (DNN) model was implemented to generate a probability map for predicting the position of tumors in both lung and liver cases.^63^ The accuracy of the model was evaluated by calculating the Euclidian distance in 3D space between the predicted and actual tumor positions. The results showed an overall mean accuracy of 1.6 ± 0.7 mm for all patients with a computation time of less than 40 ms. An encoder‐decoder CNN architecture was trained to verify the accuracy of tumor contouring with minimal mistracking caused by bone structures using DRRs.^60^ The calculated tracking error using the results of the conventional template‐matching method was approximately 1 mm with a short processing time of 25 ms/frame for contouring and tracking. The results of these studies show that AI‐based tracking is a practical approach due to its faster processing time compared to traditional methods (e.g., respiratory gating). This conforms to the American Association of Physicists in Medicine (AAPM) recommendation that real‐time tracking should have a total latency of less than 500 ms.^140^ Whereas, in respiratory gating, the radiation beam is on only when the intended target is within the gating window. This extends the delivery time as the radiation beam is not continuously on.^18^ A study performed to measure the delay time of two different linacs, Edge (Varian Medical System, Inc, USA) and Versa‐HD (Elekta Instrument AB Stockholm, Sweden), found that the beam‐on time was approximately 303  ±  45  and 1664  ±  72 ms for Edge and Versa‐HD, respectively. The beam‐off time delay was approximately 34  ±  25 ms for Edge and 25  ±  30 ms for Versa‐HD.^141^

Second, AI‐based models can also predict tumor motion patterns based on previous data, allowing for more personalized treatment plans for each patient. This can be achieved by continuously monitoring tumor positions and correcting patient setup errors through the analysis of image registration between images from various treatment fractions. Image registration methods involve a quality metric that quantifies the registration accuracy and an optimizer that adjusts the geometric transformation parameters to maximize the quality metric.^142^ However, as the complexity and degree of freedom increase, more powerful optimization algorithms (e.g., deep learning‐based registration) are required to capture and analyze all the complexities. Abbasi et al.,^143^ for example, showed that a deep unsupervised CNN model based on the co‐registration of CT/MR brain images can be clinically applicable due to reasonable registration time (203 ms) and higher accuracy compared to affine registration.

The results of these simulated models have all supported the feasibility of robust real‐time tumor tracking with AI and paved the way for the integration of AI technologies into clinical practice, improving treatment outcomes for cancer patients.

### 5.2 Transition to deep learning

As illustrated in Figure 1, there has been a shift in AI‐based motion‐tracking research from ML to DL over the past decade. The drivers of this transition include the presence of large, high‐quality, publicly available labeled datasets, along with the rapid advances in parallel graphic processing unit (GPU) computing, enabling more time‐efficient computing and image analysis.^61^, ^144^ Before the emergence of powerful computers, CNNs, as a component of larger DL networks, required a significant amount of time to make predictions and provide results when using a central processing unit (CPU) (Table 1).^58^ Classic ML techniques require greater human input to achieve reasonable results, intrinsically introducing human error and bias that may influence a study's outcome.^145^ For instance, in classic ML workflow, the feature selection has a major impact on the performance of ML models. They are usually selected manually and then models are built based on those features to categorize the object in the image. Biases in feature selection can result in incorrect discrimination between classes.^145^ With DL, the extraction of the relevant features from images and modeling steps is automatic.^120^, ^144^ Compared with classic ML approaches, DL‐based methods are more generalizable and robust, as the same network and architecture used for one image modality can be applied to different pairs of image modalities with minimal adjustments.^26^, ^125^ This enables fast translation to multiple clinically useful imaging modalities. Unlike classic ML methods that tend to reach a plateau at a certain level of performance when more examples and training data are added to the network,^146^ DL networks often continue to improve as the size of data increases.^120^ DL‐based methods have gained significant research and clinical interest in medical imaging generally and radiotherapy particularly due to these specific advantages.

### 5.3 Current limitations and potential solutions

Despite the proliferation of AI‐based solutions in real‐time tumor motion management in radiotherapy, there are some challenges that need to be addressed. One of the main challenges is the lack of standardized protocols for integrating AI into clinical practice.^147^, ^148^ As AI algorithms become more sophisticated and capable of analyzing complex data, there is a need for guidelines on how to incorporate AI into the treatment process effectively.

There are concerns about the reliability, accuracy, and generalizability of AI algorithms in predicting tumor motion as the presence of bias in human‐led data collection and model training, coupled with a lack of standardized reporting for research such as feature selection, training, validation, and testing: even number of cases often go unreported by many authors make it difficult to apply these models in practice.^149^ Moreover, trained algorithms often fail when applied to different datasets due to limited generalization (resulting from small and homogeneous training data sets).^146^, ^150^ The impact of such variations commonly limits model accuracy and generalizability. A comprehensive summary of the works related to AI‐based target tracking in three specific regions of the body: thoracic, abdomen, and pelvic are presented in the form of three tables (Tables 1, 2, 3). They provide an in‐depth analysis of various aspects related to the dataset, network, input, tracking targets, key research findings, and the year of publication. According to these tables, some studies had a small sample size, which led to insufficient patient datasets for a thorough analysis of intrafraction motion. For instance, Isaksson et al.^41^ included three patients, Yan et al.,^44^ and Cui et al.^23^ used four and five patients, respectively. Small sample sizes can produce false‐negative results^151^ and may yield falsely higher accuracy due to overfitting or random effects.^146^, ^152^ Ultimately, the limited size of the training and test sets introduces bias and increases variance in model performance.^115^, ^120^ To resolve this issue, many authors follow Cohen's equations^153^ to determine the effect size by calculating the mean and variance. However, there are two methods to estimate the variance: (a) population variance, and (b) sample variance. To calculate population variance, all data is needed, whereas for computing sample variance, we only need a portion of it. This difference in variance calculation can affect the outcome of these measurements. To resolve this issue, Rajput et al.^146^ proposed a new method to evaluate sample size in ML studies by combining the effect sizes and ML accuracy. The authors believe that these practical criteria can serve as a reference for both the authors and editors to evaluate the adequacy of the selected sample size for a study.

Recently, data augmentation has been commonly used in ML, especially for DL models, where large and diverse datasets are essential for good performance. For example, a study conducted by Terunuma et al.^60^ had only four patients for their study, which was not enough to train their deep‐learning model. To tackle this issue, they generated separate a soft‐tissue DRR and a bone‐structure DRR from a single patient's 3D CT data. This approach allowed them to produce a large number of training images, which were essential for the effective training of their DL model. Despite the advantages of using augmentation, this technique can limit the model's efficiency. These augmentation algorithms work optimally when both the training and testing data are derived from the same distribution. If this assumption is not valid, the likelihood of these methods being useful is very low.^154^

The majority of authors have opted for using 2D images as input files in their studies, mainly due to the shorter calculation time required. A major challenge is that the two‐dimensional (2D) kV images that are acquired from Electronic Portal Imaging Device (EPID) panels or flat panel detectors are rarely able to continuously track the tumor due to the inferior tumor visibility on 2D kV images. Therefore, it is essential to develop robust algorithms that are capable of handling artifacts, noise, and scatter to improve image quality for accurate and reliable detection. Also, the estimation of 3D tumor position is based on only 2D imaging information, ultimately affecting the overall precision and efficacy of the model, particularly if there are inter‐fractional changes in the positions of the tumor and organs. Ahmed et al.^32^ calculated 3D ground truth from the triangulation of marker position in orthogonally paired kV/MV images. Due to the low visibility of the markers in the MV field of view, the calculation of triangulated positions for all kV images was not possible. Hirai et al.^63^ used cropped images as input files and derived the 3D position of the tumor from the 2D tumor position. Their results indicated tracking accuracy in the lateral direction was significantly degraded.

Furthermore, each treatment site consists of complex and interconnected anatomical structures. For example, the abdomen area includes the liver, stomach, pancreatic, intestines, blood vessels, etc. Distinguishing between abnormalities and normal structures requires advanced image processing techniques and feature extraction methods. Furthermore, abnormalities can appear in various shapes and sizes, making the task even more challenging. To handle this complexity, computer vision algorithms must be capable of comprehending the context and relationships between different structures within the studied treatment regions.^155^

In addition, there are ethical considerations surrounding the use of AI in healthcare, including issues related to patient privacy, data security, and especially determining the level of human involvement in decision‐making processes. As AI becomes more integrated into clinical practice, it is essential to establish clear guidelines on the roles and responsibilities of healthcare professionals and AI algorithms in patient care. This makes it important to safeguard patient information and comply with healthcare regulations.

In 2020, Mongan et al.^150^ proposed a comprehensive guideline for the evaluation and development of reliable AI models in medical imaging (Checklist for Artificial Intelligence in Medical Imaging, CLAIM). The CLAIM framework details 42 items that guide authors and reviewers of AI manuscripts by providing recommendations on generalizability and reproducibility for frequently encountered tasks like classification, image reconstruction, image analysis, and workflow optimization. Unfortunately, a small proportion of AI studies fulfill key items in CLAIM guidelines within their methods and results sections.^156^, ^157^ We recommend that authors and reviewers have a solid understanding of relevant reporting guidelines, ensuring essential elements are adequately reported in manuscripts for publication. Improved CLAIM adherence could enhance the robustness and generalizability of trained algorithms to boost the adoption of AI in clinical practice and accelerate investigators' progress toward future innovation.

### 5.4 Future direction

It is important to highlight that the use of artificial intelligence in monitoring intrafraction motion during radiotherapy is an area that has not been extensively researched. Also, a lack of transparency among several papers reporting the patients’ outcomes has been observed. This implies that there is a dearth of literature on the potential benefits and challenges of using AI for this purpose. To address this gap, more comprehensive research needs to be conducted in this field to evaluate the efficacy and safety of AI‐powered tools in improving radiotherapy outcomes. Also, it is important to study the effects of various factors on tracking accuracy, such as motion range, tumor position, and patient size. By conducting such investigations, we can predict tracking accuracy based on patient characteristics and develop patient selection criteria.

Furthermore, the current AI methods require powerful computers to perform the calculation in a short amount of time. This high latency issue can be a considerable obstacle in achieving real‐time monitoring of intrafraction motion during radiotherapy. This can be solved by improving the IT infrastructure in clinics. By upgrading the hardware and software components, clinics can improve the processing speed of AI algorithms, thus enabling more accurate and timely monitoring of intrafraction motion during radiotherapy.

Looking toward the future, the role of AI in real‐time tumor motion management in radiotherapy is likely to continue to evolve. Advancements in AI technologies, especially deep learning, will further improve the accuracy and efficiency of tumor targeting during radiotherapy treatments. Additionally, the integration of AI into other aspects of cancer care, such as treatment planning and monitoring, will allow for more personalized and effective treatment strategies.

However, it is essential to understand that AI cannot replace human medical professionals. Its role is to support and enhance the capabilities of healthcare providers. Before implementing AI systems in clinical settings, it is essential to evaluate their safety and reliability thoroughly. This evaluation process is crucial to ensure that AI‐powered tools are stable, secure, and not prone to errors that could put patients' health at risk. To achieve this, training and education of clinical staff is necessary to enable them to effectively utilize and integrate AI‐powered tools into their clinical practices.

## 6 CONCLUSION

The use of AI in motion monitoring during radiotherapy has demonstrated significant success in improving accuracy compared to conventional techniques. AI methods have shown potential in markerless tracking of intrafraction movements by enhancing target visibility within onboard projection images. Although AI networks can provide accurate predictions, their decisions can be difficult to interpret, explain, debug, and validate, which poses regulatory and ethical challenges. Further research is necessary to enhance the calculation efficiency, accuracy, and robustness of AI models for intrafraction motion management, as well as to reliably evaluate the performance of proposed AI methods.

## AUTHOR CONTRIBUTIONS

Elahheh Salari and Xiaofeng Yang contributed to the article's conception design, and interpretation of the relevant literature, and wrote the review paper. Jing Wang, Jacob F. Wynne, and Chih‐Wei Chang, Yizhou Wu contributed to the conception, and design, and wrote the review paper.

## CONFLICT OF INTEREST STATEMENT

The authors declare no conflicts of interest.

## References

[1] Parsai EI , Pearson D , Kvale T . Consequences of removing the flattening filter from linear accelerators in generating high dose rate photon beams for clinical applications: a Monte Carlo study verified by measurement. Nucl Instrum Methods Phys Res Sect B. 2007;261(1):755‐759.

[2] Ghemiş DM , Marcu LG . Progress and prospects of flattening filter free beam technology in radiosurgery and stereotactic body radiotherapy. Crit Rev Oncol Hematol. 2021;163:103396. 34146680 10.1016/j.critrevonc.2021.103396

[3] Lai Y , Chen S , Xu C , et al. Dosimetric superiority of flattening filter free beams for single‐fraction stereotactic radiosurgery in single brain metastasis. Oncotarget. 2017;8(21):35272‐35279. 27823985 10.18632/oncotarget.13085 PMC5471053

[4] Mamballikalam G , Senthilkumar S , Clinto CO , et al. Time motion study to evaluate the impact of flattening filter free beam on overall treatment time for frameless intracranial radiosurgery using Varian TrueBeam((R)) linear accelerator. Rep Pract Oncol Radiother. 2021;26(1):111‐118. 34046221 10.5603/RPOR.a2021.0018 PMC8149126

[5] Lovelock DM , Messineo AP , Cox BW , Kollmeier MA , Zelefsky MJ . Continuous monitoring and intrafraction target position correction during treatment improves target coverage for patients undergoing SBRT prostate therapy. Int J Radiat Oncol Biol Phys. 2015;91(3):588‐594. 25680601 10.1016/j.ijrobp.2014.10.049

[6] Fast M , van de Schoot A , van de Lindt T , Carbaat C , van der Heide U , Sonke JJ . Tumor trailing for liver SBRT on the MR‐Linac. Int J Radiat Oncol Biol Phys. 2019;103(2):468‐478. 30243573 10.1016/j.ijrobp.2018.09.011

[7] Lei Y , Tian Z , Wang T , et al. Deep learning‐based real‐time volumetric imaging for lung stereotactic body radiation therapy: a proof of concept study. Phys Med Biol. 2020;65(23):235003. 33080578 10.1088/1361-6560/abc303 PMC11756341

[8] Han B , Wu B , Hu F , et al. Simulation of dosimetric consequences of intrafraction variation of tumor drift in lung cancer stereotactic body radiotherapy. Front Oncol. 2022;12:1010411. 36891502 10.3389/fonc.2022.1010411 PMC9987420

[9] Boda‐Heggemann J , Köhler FM , Wertz H , et al. Intrafraction motion of the prostate during an IMRT session: a fiducial‐based 3D measurement with Cone‐beam CT. Radiat Oncol. 2008;3(1):37. 18986517 10.1186/1748-717X-3-37 PMC2588616

[10] Poulsen PR , Worm ES , Hansen R , Larsen LP , Grau C , Høyer M . Respiratory gating based on internal electromagnetic motion monitoring during stereotactic liver radiation therapy: first results. Acta Oncol. 2015;54(9):1445‐1452. 26198651 10.3109/0284186X.2015.1062134

[11] Tacke MB , Nill S , Krauss A , Oelfke U . Real‐time tumor tracking: automatic compensation of target motion using the Siemens 160 MLC. Med Phys. 2010;37(2):753‐761. 20229885 10.1118/1.3284543

[12] Jöhl A , Ehrbar S , Guckenberger M , et al. The ideal couch tracking system—requirements and evaluation of current systems. J Appl Clin Med Phys. 2019;20(10):152‐159. 10.1002/acm2.12731 PMC6806475 31535782

[13] Bottero M , Dipasquale G , Lancia A , Miralbell R , Jaccard M , Zilli T . Electromagnetic transponder localization and real‐time tracking for prostate cancer radiation therapy: clinical impact of metallic hip prostheses. Pract Radiat Oncol. 2020;10(6):e538‐e542. 32201320 10.1016/j.prro.2020.03.003

[14] Boggs DH , Popple R , McDonald A , et al. Electromagnetic transponder based tracking and gating in the radiotherapeutic treatment of thoracic malignancies. Pract Radiat Oncol. 2019;9(6):456‐464. 31283991 10.1016/j.prro.2019.06.021

[15] Zollner B , Heinz C , Pitzler S , et al. Stereoscopic X‐ray imaging, cone beam CT, and couch positioning in stereotactic radiotherapy of intracranial tumors: preliminary results from a cross‐modality pilot installation. Radiat Oncol. 2016;11(1):158. 27927235 10.1186/s13014-016-0735-2 PMC5142336

[16] Lyatskaya Y , Lu HM , Chin L . Performance and characteristics of an IR localizing system for radiation therapy. J Appl Clin Med Phys. 2006;7(2):18‐37. 17533324 10.1120/jacmp.v7i2.2190 PMC5722449

[17] Western C , Hristov D , Schlosser J . Ultrasound imaging in radiation therapy: from interfractional to intrafractional guidance. Cureus. 2015;7(6):e280. 26180704 10.7759/cureus.280 PMC4494460

[18] Bertholet J , Knopf A , Eiben B , et al. Real‐time intrafraction motion monitoring in external beam radiotherapy. Phys Med Biol. 2019;64(15):15tr01. 10.1088/1361-6560/ab2ba8 PMC7655120 31226704

[19] Wu VWC , Ng APL , Cheung EKW . Intrafractional motion management in external beam radiotherapy. J Xray Sci Technol. 2019;27(6):1071‐1086. 31476194 10.3233/XST-180472

[20] Li G , Zhang X , Song X , et al. Machine learning for predicting accuracy of lung and liver tumor motion tracking using radiomic features. Quant Imaging Med Surg. 2023;13(3):1605‐1618. 36915317 10.21037/qims-22-621 PMC10006135

[21] Zhang X , Song X , Li G , et al. Machine learning radiomics model for external and internal respiratory motion correlation prediction in lung tumor. Technol Cancer Res Treat. 2022;21:15330338221143224. 36476136 10.1177/15330338221143224 PMC9742719

[22] Yun J , Mackenzie M , Rathee S , Robinson D , Fallone BG . An artificial neural network (ANN)‐based lung‐tumor motion predictor for intrafractional MR tumor tracking. Med Phys. 2012;39(7):4423‐4433. 22830775 10.1118/1.4730294

[23] Cui Y , Dy JG , Alexander B , Jiang SB . Fluoroscopic gating without implanted fiducial markers for lung cancer radiotherapy based on support vector machines. Phys Med Biol. 2008;53(16):N315‐N327. 18660557 10.1088/0031-9155/53/16/N01

[24] Lin H , Zou W , Li T , Feigenberg SJ , Teo BK , Dong L . A super‐learner model for tumor motion prediction and management in radiation therapy: development and feasibility evaluation. Sci Rep. 2019;9(1):14868. 31619736 10.1038/s41598-019-51338-y PMC6795883

[25] Mori S . Deep architecture neural network‐based real‐time image processing for image‐guided radiotherapy. Phys Med. 2017;40:79‐87. 28743618 10.1016/j.ejmp.2017.07.013

[26] Terpstra ML , Maspero M , Bruijnen T , Verhoeff JJC , Lagendijk JJW , van den Berg CAT . Real‐time 3D motion estimation from undersampled MRI using multi‐resolution neural networks. Med Phys. 2021;48(11):6597‐6613. 34525223 10.1002/mp.15217 PMC9298075

[27] Teo PT , Bajaj A , Randall J , Lou B , Shah J , Gopalakrishnan M , et al. Deterministic small‐scale undulations of image‐based risk predictions from the deep learning of lung tumors in motion. Med Phys. 2022;49(11):7347‐7356. 35962958 10.1002/mp.15869 PMC10115400

[28] Ronneberger O , Fischer P , Brox T . U‐Net: convolutional networks for biomedical image segmentation. Medical Image Computing and Computer‐Assisted Intervention—MICCAI. 2015;2015:234‐241.

[29] Pohl M , Uesaka M , Demachi K . Bhusal Chhatkuli R. Prediction of the motion of chest internal points using a recurrent neural network trained with real‐time recurrent learning for latency compensation in lung cancer radiotherapy. Comput Med Imaging Graph. 2021;91:101941. 34265553 10.1016/j.compmedimag.2021.101941

[30] Momin S , Lei Y , Tian Z , et al. Lung tumor segmentation in 4D CT images using motion convolutional neural networks. Med Phys. 2021;48(11):7141‐7153. 34469001 10.1002/mp.15204 PMC11700498

[31] Bharadwaj S , Prasad S , Almekkawy M . An upgraded Siamese neural network for motion tracking in ultrasound image sequences. IEEE Trans Ultrason Ferroelectr Freq Control. 2021;68(12):3515‐3527. 34232873 10.1109/TUFFC.2021.3095299

[32] Ahmed AM , Gargett M , Madden L , et al. Evaluation of deep learning based implanted fiducial markers tracking in pancreatic cancer patients. Biomed Phys Eng Express. 2023;9(3). 10.1088/2057-1976/acb550 36689758

[33] Wang G , Li Z , Li G , et al. Real‐time liver tracking algorithm based on LSTM and SVR networks for use in surface‐guided radiation therapy. Radiat Oncol. 2021;16(1):13. 33446245 10.1186/s13014-020-01729-7 PMC7807524

[34] Ma Y , Mao J , Liu X , et al. Deep learning‐based internal gross target volume definition in 4D CT images of lung cancer patients. Med Phys. 2023;50(4):2303‐2316. 36398404 10.1002/mp.16106

[35] Page MJ , McKenzie JE , Bossuyt PM , et al. The PRISMA 2020 statement: an updated guideline for reporting systematic reviews. PLoS Med. 2021;18(3):e1003583. 33780438 10.1371/journal.pmed.1003583 PMC8007028

[36] Siegel RL , Miller KD , Fuchs HE , Jemal A . Cancer statistics, 2022. CA Cancer J Clin. 2022;72(1):7‐33. 35020204 10.3322/caac.21708

[37] Huang Y , Dong Z , Wu H , Li C , Liu H , Zhang Y . Deep learning‐based synthetization of real‐time in‐treatment 4D images using surface motion and pretreatment images: a proof‐of‐concept study. Med Phys. 2022;49(11):7016‐7024. 35833590 10.1002/mp.15858

[38] Molitoris JK , Diwanji T . Advances in the use of motion management and image guidance in radiation therapy treatment for lung cancer. J Thorac Dis. 2018;10(21):S2437‐S2450. Suppl. 30206490 10.21037/jtd.2018.01.155 PMC6123191

[39] Cole AJ , Hanna GG , Jain S , O'Sullivan JM . Motion management for radical radiotherapy in non‐small cell lung cancer. Clin Oncol (R Coll Radiol). 2014;26(2):67‐80. 24290238 10.1016/j.clon.2013.11.001

[40] Liu H , Chen R , Tong C , Liang XW . MRI versus CT for the detection of pulmonary nodules: a meta‐analysis. Medicine (Baltimore). 2021;100(42):e27270. 34678861 10.1097/MD.0000000000027270 PMC8542155

[41] Isaksson M , Jalden J , Murphy MJ . On using an adaptive neural network to predict lung tumor motion during respiration for radiotherapy applications. Med Phys. 2005;32(12):3801‐3809. 16475780 10.1118/1.2134958

[42] Kakar M , Nyström H , Aarup LR , Nøttrup TJ , Olsen DR . Respiratory motion prediction by using the adaptive neuro fuzzy inference system (ANFIS). Phys Med Biol. 2005;50(19):4721. 16177500 10.1088/0031-9155/50/19/020

[43] Murphy MJ , Dieterich S . Comparative performance of linear and nonlinear neural networks to predict irregular breathing. Phys Med Biol. 2006;51(22):5903‐5914. 17068372 10.1088/0031-9155/51/22/012

[44] Yan H , Yin FF , Zhu GP , Ajlouni M , Kim JH . Adaptive prediction of internal target motion using external marker motion: a technical study. Phys Med Biol. 2006;51(1):31‐44. 16357429 10.1088/0031-9155/51/1/003

[45] Zhang Q , Pevsner A , Hertanto A , et al. A patient‐specific respiratory model of anatomical motion for radiation treatment planning. Med Phys. 2007;34(12):4772‐4781. 18196805 10.1118/1.2804576

[46] Lin T , Cervino LI , Tang X , Vasconcelos N , Jiang SB . Fluoroscopic tumor tracking for image‐guided lung cancer radiotherapy. Phys Med Biol. 2009;54(4):981‐992. 19147898 10.1088/0031-9155/54/4/011

[47] Lin T , Li R , Tang X , Dy JG , Jiang SB . Markerless gating for lung cancer radiotherapy based on machine learning techniques. Phys Med Biol. 2009;54(6):1555‐1563. 19229098 10.1088/0031-9155/54/6/010

[48] Riaz N , Shanker P , Wiersma R , et al. Predicting respiratory tumor motion with multi‐dimensional adaptive filters and support vector regression. Phys Med Biol. 2009;54(19):5735‐5748. 19729711 10.1088/0031-9155/54/19/005 PMC12165777

[49] Torshabi AE , Pella A , Riboldi M , Baroni G . Targeting accuracy in real‐time tumor tracking via external surrogates: a comparative study. Technol Cancer Res Treat. 2010;9(6):551‐562. 21070077 10.1177/153303461000900603

[50] Cervino LI , Du J , Jiang SB . MRI‐guided tumor tracking in lung cancer radiotherapy. Phys Med Biol. 2011;56(13):3773‐3785. 21628775 10.1088/0031-9155/56/13/003

[51] Krauss A , Nill S , Oelfke U . The comparative performance of four respiratory motion predictors for real‐time tumour tracking. Phys Med Biol. 2011;56(16):5303‐5317. 21799237 10.1088/0031-9155/56/16/015

[52] Li R , Lewis JH , Jia X , et al. On a PCA‐based lung motion model. Phys Med Biol. 2011;56(18):6009‐6030. 21865624 10.1088/0031-9155/56/18/015 PMC3915048

[53] Fayad H , Pan T , Pradier O , Visvikis D . Patient specific respiratory motion modeling using a 3D patient's external surface. Med Phys. 2012;39(6):3386‐3395. 22755719 10.1118/1.4718578 PMC4032399

[54] Torshabi AE . Investigation of the robustness of adaptive neuro‐fuzzy inference system for tracking moving tumors in external radiotherapy. Australas Phys Eng Sci Med. 2014;37(4):771‐778. 25412886 10.1007/s13246-014-0313-6

[55] Li G , Wei J , Huang H , Gaebler CP , Yuan A , Deasy JO . Automatic assessment of average diaphragm motion trajectory from 4DCT images through machine learning. Biomed Phys Eng Express. 2015;1(4):045015. 27110388 10.1088/2057-1976/1/4/045015 PMC4840474

[56] Bukhari W , Hong SM . Real‐time prediction and gating of respiratory motion using an extended Kalman filter and Gaussian process regression. Phys Med Biol. 2015;60(1):233‐252. 25489980 10.1088/0031-9155/60/1/233

[57] Yun J , Yip E , Gabos Z , Wachowicz K , Rathee S , Fallone BG . Neural‐network based autocontouring algorithm for intrafractional lung‐tumor tracking using Linac‐MR. Med Phys. 2015;42(5):2296‐2310. 25979024 10.1118/1.4916657

[58] Bukovsky I , Homma N , Ichiji K , et al. A fast neural network approach to predict lung tumor motion during respiration for radiation therapy applications. Biomed Res Int. 2015;2015:489679. 25893194 10.1155/2015/489679 PMC4393907

[59] Park S , Lee SJ , Weiss E , Motai Y . Intra‐ and inter‐fractional variation prediction of lung tumors using fuzzy deep learning. IEEE J Transl Eng Health Med. 2016;4:4300112. 27170914 10.1109/JTEHM.2016.2516005 PMC4862314

[60] Teo TP , Ahmed SB , Kawalec P , et al. Feasibility of predicting tumor motion using online data acquired during treatment and a generalized neural network optimized with offline patient tumor trajectories. Med Phys. 2018;45(2):830‐845. 29244902 10.1002/mp.12731

[61] Terunuma T , Tokui A , Sakae T . Novel real‐time tumor‐contouring method using deep learning to prevent mistracking in X‐ray fluoroscopy. Radiol Phys Technol. 2018;11(1):43‐53. 29285686 10.1007/s12194-017-0435-0 PMC5840203

[62] Edmunds D , Sharp G , Winey B . Automatic diaphragm segmentation for real‐time lung tumor tracking on cone‐beam CT projections: a convolutional neural network approach. Biomed Phys Eng Express. 2019;5(3):035005. 34234960 10.1088/2057-1976/ab0734 PMC8260092

[63] Jiang K , Fujii F , Shiinoki T . Prediction of lung tumor motion using nonlinear autoregressive model with exogenous input. Phys Med Biol. 2019;64(21):21NT02. 10.1088/1361-6560/ab49ea 31574490

[64] Hirai R , Sakata Y , Tanizawa A , Mori S . Real‐time tumor tracking using fluoroscopic imaging with deep neural network analysis. Phys Med. 2019;59:22‐29. 30928062 10.1016/j.ejmp.2019.02.006

[65] Wei R , Zhou F , Liu B , et al. Real‐time tumor localization with single x‐ray projection at arbitrary gantry angles using a convolutional neural network (CNN). Phys Med Biol. 2020;65(6):065012. 31896093 10.1088/1361-6560/ab66e4

[66] Mori S , Hirai R , Sakata Y . Simulated four‐dimensional CT for markerless tumor tracking using a deep learning network with multi‐task learning. Phys Med. 2020;80:151‐158. 33189045 10.1016/j.ejmp.2020.10.023

[67] Wang C , Hunt M , Zhang L , et al. Technical Note: 3D localization of lung tumors on cone beam CT projections via a convolutional recurrent neural network. Med Phys. 2020;47(3):1161‐1166. 31899807 10.1002/mp.14007 PMC7067648

[68] Sakata Y , Hirai R , Kobuna K , Tanizawa A , Mori S . A machine learning‐based real‐time tumor tracking system for fluoroscopic gating of lung radiotherapy. Phys Med Biol. 2020;65(8):085014. 32097899 10.1088/1361-6560/ab79c5

[69] Dai X , Lei Y , Roper J , Chen Y , et al. Deep learning‐based motion tracking using ultrasound images. Med Phys. 2021;48(12):7747‐7756. 34724712 10.1002/mp.15321 PMC11742242

[70] He X , Cai W , Li F , et al. Decompose kV projection using neural network for improved motion tracking in paraspinal SBRT. Med Phys. 2021;48(12):7590‐7601. 34655442 10.1002/mp.15295 PMC9454326

[71] Liu C , Wang Q , Si W , Ni X . NuTracker: a coordinate‐based neural network representation of lung motion for intrafraction tumor tracking with various surrogates in radiotherapy. Phys Med Biol. 2022;68(1):aca873. 10.1088/1361-6560/aca873 36537890

[72] Lombardo E , Rabe M , Xiong Y , et al. Offline and online LSTM networks for respiratory motion prediction in MR‐guided radiotherapy. Phys Med Biol. 2022;67(9):ac60b7. 10.1088/1361-6560/ac60b7 35325880

[73] Hindley N , Shieh CC , Keall P . A patient‐specific deep learning framework for 3D motion estimation and volumetric imaging during lung cancer radiotherapy. Phys Med Biol. 2023;68(14):ace1d0. 10.1088/1361-6560/ace1d0 37364571

[74] Huttinga NRF , Akdag O , Fast MF , et al. Real‐time myocardial landmark tracking for MRI‐guided cardiac radio‐ablation using Gaussian Processes. Phys Med Biol. 2023;68(14):ace023. 10.1088/1361-6560/ace023 37339638

[75] Lombardo E , Rabe M , Xiong Y , et al. Evaluation of real‐time tumor contour prediction using LSTM networks for MR‐guided radiotherapy. Radiother Oncol. 2023;182:109555. 36813166 10.1016/j.radonc.2023.109555

[76] Zhou D , Nakamura M , Mukumoto N , Matsuo Y , Mizowaki T . Feasibility study of deep learning‐based markerless real‐time lung tumor tracking with orthogonal X‐ray projection images. J Appl Clin Med Phys. 2023;24(4):e13894. 36576920 10.1002/acm2.13894 PMC10113683

[77] Liang Z , Zhang M , Shi C , Huang ZR . Real‐time respiratory motion prediction using photonic reservoir computing. Sci Rep. 2023;13(1):5718. 37029184 10.1038/s41598-023-31296-2 PMC10082218

[78] Zhang J , Wang Y , Bai X , Chen M . Extracting lung contour deformation features with deep learning for internal target motion tracking: a preliminary study. Phys Med Biol. 2023;68(19):acf10e. 10.1088/1361-6560/acf10e 37586388

[79] Dai J , Dong G , Zhang C , et al. Volumetric tumor tracking from a single cone‐beam X‐ray projection image enabled by deep learning. Med Image Anal. 2024;91:102998. 37857066 10.1016/j.media.2023.102998

[80] Fu Y , Zhang P , Fan Q , et al. Deep learning‐based target decomposition for markerless lung tumor tracking in radiotherapy. Med Phys. 2024;51(6):4271‐4282. 38507259 10.1002/mp.17039 PMC12123686

[81] Abbas H , Chang B , Chen ZJ . Motion management in gastrointestinal cancers. J Gastrointest Oncol. 2014;5(3):223‐235. 24982771 10.3978/j.issn.2078-6891.2014.028 PMC4074952

[82] Goyal S , Rangankar V , Deshmukh S , Prabhu A, SJ . MRI evaluation of soft tissue tumors and tumor‐like lesions of extremities. Cureus. 2023;15(4):e37047. 37153328 10.7759/cureus.37047 PMC10154641

[83] Gou S , Lee P , Hu P , Rwigema JC , Sheng K . Feasibility of automated 3‐dimensional magnetic resonance imaging pancreas segmentation. Adv Radiat Oncol. 2016;1(3):182‐193. 27868105 10.1016/j.adro.2016.05.002 PMC5113135

[84] Stemkens B , Tijssen RH , de Senneville BD , Lagendijk JJ , van den Berg CA . Image‐driven, model‐based 3D abdominal motion estimation for MR‐guided radiotherapy. Phys Med Biol. 2016;61(14):5335‐5355. 27362636 10.1088/0031-9155/61/14/5335

[85] Dick D , Wu X , Hatoum GF , Zhao W . A fiducial‐less tracking method for radiation therapy of liver tumors by diaphragm disparity analysis part 2: validation study by using clinical data. J Radiat Oncol. 2018;7(4):345‐356.

[86] Huang P , Su L , Chen S , et al. 2D ultrasound imaging based intra‐fraction respiratory motion tracking for abdominal radiation therapy using machine learning. Phys Med Biol. 2019;64(18):185006. 31323649 10.1088/1361-6560/ab33db

[87] Huang P , Yu G , Lu H , et al. Attention‐aware fully convolutional neural network with convolutional long short‐term memory network for ultrasound‐based motion tracking. Med Phys. 2019;46(5):2275‐2285. 30912590 10.1002/mp.13510

[88] Zhao W , Shen L , Han B , et al. Markerless pancreatic tumor target localization enabled by deep learning. Int J Radiat Oncol Biol Phys. 2019;105(2):432‐439. 31201892 10.1016/j.ijrobp.2019.05.071 PMC6732032

[89] Liang Z , Zhou Q , Yang J , et al. Artificial intelligence‐based framework in evaluating intrafraction motion for liver cancer robotic stereotactic body radiation therapy with fiducial tracking. Med Phys. 2020;47(11):5482‐5489. 32996131 10.1002/mp.14501

[90] Liu M , Cygler JE , Vandervoort E. Patient‐specific PTV margins for liver stereotactic body radiation therapy determined using support vector classification with an early warning system for margin adaptation. Med Phys. 2020;47(10):5172‐5182. 32740935 10.1002/mp.14419

[91] Roggen T , Bobic M , Givehchi N , Scheib SG . Deep learning model for markerless tracking in spinal SBRT. Phys Med. 2020;74:66‐73. 32422577 10.1016/j.ejmp.2020.04.029

[92] Terpstra ML , Maspero M , dʼAgata F , et al. Deep learning‐based image reconstruction and motion estimation from undersampled radial k‐space for real‐time MRI‐guided radiotherapy. Phys Med Biol. 2020;65(15):155015. 32408295 10.1088/1361-6560/ab9358

[93] Romaguera LV , Mezheritsky T , Mansour R , Carrier JF , Kadoury S . Probabilistic 4D predictive model from in‐room surrogates using conditional generative networks for image‐guided radiotherapy. Med Image Anal. 2021;74:102250. 34601453 10.1016/j.media.2021.102250

[94] Shao HC , Huang X , Folkert MR , Wang J , Zhang Y . Automatic liver tumor localization using deep learning‐based liver boundary motion estimation and biomechanical modeling (DL‐Bio). Med Phys. 2021;48(12):7790‐7805. 34632589 10.1002/mp.15275 PMC8678353

[95] Liu L , Shen L , Johansson A , et al. Real time volumetric MRI for 3D motion tracking via geometry‐informed deep learning. Med Phys. 2022;49(9):6110‐6119. 35766221 10.1002/mp.15822 PMC10323755

[96] Shao HC , Li T , Dohopolski MJ , et al. Real‐time MRI motion estimation through an unsupervised k‐space‐driven deformable registration network (KS‐RegNet). Phys Med Biol. 2022;67(13):ac762c. 10.1088/1361-6560/ac762c PMC9309029 35667374

[97] Shao HC , Wang J , Bai T , et al. Real‐time liver tumor localization via a single x‐ray projection using deep graph neural network‐assisted biomechanical modeling. Phys Med Biol. 2022;67(11):ac6b7b. 10.1088/1361-6560/ac6b7b PMC9233941 35483350

[98] Dai Z , He Q , Zhu L , et al. Automatic prediction model for online diaphragm motion tracking based on optical surface monitoring by machine learning. Quant Imaging Med Surg. 2023;13(4):2065‐2080. 37064379 10.21037/qims-22-242 PMC10102745

[99] Hunt B , Gill GS , Alexander DA , et al. Fast deformable image registration for real‐time target tracking during radiation therapy using Cine MRI and deep learning. Int J Radiat Oncol Biol Phys. 2023;115(4):983‐993. 36309075 10.1016/j.ijrobp.2022.09.086

[100] Shao HC , Li Y , Wang J , Jiang SB , Zhang Y . Real‐time liver tumor localization via combined surface imaging and a single x‐ray projection. Phys Med Biol. 2023;68(6):065002. 10.1088/1361-6560/acb889 PMC10394117 36731143

[101] Weng J , Bhupathiraju SHV , Samant T , Dresner A , Wu J , Samant SS . Convolutional LSTM model for cine image prediction of abdominal motion. Phys Med Biol. 2024;69(8):ad3722. 10.1088/1361-6560/ad3722 38518378

[102] Xiao H , Han X , Zhi S , et al. Ultra‐fast multi‐parametric 4D‐MRI image reconstruction for real‐time applications using a downsampling‐invariant deformable registration (D2R) model. Radiother Oncol. 2023;189:109948. 37832790 10.1016/j.radonc.2023.109948

[103] Fransson S , Tilly D , Ahnesjö A , Nyholm T , Strand R . Intrafractional motion models based on principal components in magnetic resonance guided prostate radiotherapy. Phys Imaging Radiat Oncol. 2021;20:17‐22. 34660917 10.1016/j.phro.2021.09.004 PMC8502906

[104] Oehler C , Roehner N , Sumila M , et al. Intrafraction prostate motion management for ultra‐hypofractionated radiotherapy of prostate cancer. Curr Oncol. 2022;29(9):6314‐6324. 36135065 10.3390/curroncol29090496 PMC9497512

[105] Tong X , Chen X , Li J , et al. Intrafractional prostate motion during external beam radiotherapy monitored by a real‐time target localization system. J Appl Clin Med Phys. 2015;16(2):5013. 26103174 10.1120/jacmp.v16i2.5013 PMC5690091

[106] Chrystall D , Mylonas A , Hewson E , et al. Deep learning enables MV‐based real‐time image guided radiation therapy for prostate cancer patients. Phys Med Biol. 2023;68(9):acc77c. 10.1088/1361-6560/acc77c 36963116

[107] Motley R , Ramachandran P , Fielding A . A feasibility study on the development and use of a deep learning model to automate real‐time monitoring of tumor position and assessment of interfraction fiducial marker migration in prostate radiotherapy patients. Biomed Phys Eng Express. 2022;8(3):ac34da. 10.1088/2057-1976/ac34da 34715689

[108] Zhao W , Han B , Yang Y , et al. Incorporating imaging information from deep neural network layers into image guided radiation therapy (IGRT). Radiother Oncol. 2019;140:167‐174. 31302347 10.1016/j.radonc.2019.06.027 PMC6814540

[109] Zhu N , Najafi M , Han B , Hancock S , Hristov D . Feasibility of image registration for ultrasound‐guided prostate radiotherapy based on similarity measurement by a convolutional neural network. Technol Cancer Res Treat. 2019;18:1533033818821964. 30803364 10.1177/1533033818821964 PMC6373996

[110] Mylonas A , Keall PJ , Booth JT , et al. A deep learning framework for automatic detection of arbitrarily shaped fiducial markers in intrafraction fluoroscopic images. Med Phys. 2019;46(5):2286‐2297. 30929254 10.1002/mp.13519

[111] Amarsee K , Ramachandran P , Fielding A , et al. Automatic detection and tracking of Marker seeds implanted in prostate cancer patients using a deep learning algorithm. J Med Phys. 2021;46(2):80‐87. 34566287 10.4103/jmp.JMP_117_20 PMC8415249

[112] Nguyen DT , Keall P , Booth J , Shieh CC , Poulsen P , O'Brien R . A real‐time IGRT method using a Kalman filter framework to extract 3D positions from 2D projections. Phys Med Biol. 2021;66(21):ac06e3. 10.1088/1361-6560/ac06e3 34062512

[113] Egmont‐Petersen M , de Ridder D , Handels H . Image processing with neural networks—a review. Pattern Recognit. 2002;35(10):2279‐2301.

[114] Courellis SH , Marmarelis VZ , eds. An artificial neural network for motion detection and speed estimation. 1990 IJCNN International Joint Conference on Neural Networks; 1990 17–21 June 1990.

[115] Way TW , Sahiner B , Hadjiiski LM , Chan H‐P . Effect of finite sample size on feature selection and classification: a simulation study. Med Phys. 2010;37(2):907‐920. 20229900 10.1118/1.3284974 PMC2826389

[116] Suthaharan S . Support vector machine. In: Suthaharan S , ed. Machine Learning Models and Algorithms for Big Data Classification: Thinking with Examples for Effective Learning. Springer US; 2016:207‐235.

[117] Gao C , Sang N , Huang R , eds. Online Transfer Boosting for object tracking. Proceedings of the 21st International Conference on Pattern Recognition (ICPR2012); 2012 11–15 Nov. 2012.

[118] Malekian A , Chitsaz N . Chapter 4 ‐ Concepts, procedures, and applications of artificial neural network models in streamflow forecasting. In: Sharma P , Machiwal D , eds. Advances in Streamflow Forecasting. Elsevier; 2021:115‐147.

[119] Park YS , Lek S . Chapter 7 ‐ Artificial neural networks: multilayer perceptron for ecological modeling. In: Jørgensen SE , ed. Developments in Environmental Modelling. Elsevier; 2016:123‐140.

[120] Castiglioni I , Rundo L , Codari M , et al. AI applications to medical images: from machine learning to deep learning. Phys Med. 2021;83:9‐24. 33662856 10.1016/j.ejmp.2021.02.006

[121] Nossier SA , Wall J , Moniri M , Glackin C , Cannings N . An experimental analysis of deep learning architectures for supervised speech enhancement. Electronics. 2021;10(1):17.

[122] Fusco R , Granata V , Grazzini G , et al. Radiomics in medical imaging: pitfalls and challenges in clinical management. Jpn J Radiol. 2022;40(9):919‐929. 35344132 10.1007/s11604-022-01271-4

[123] van Timmeren JE , Cester D , Tanadini‐Lang S , Alkadhi H , Baessler B . Radiomics in medical imaging‐“how‐to” guide and critical reflection. Insights Imaging. 2020;11(1):91. 32785796 10.1186/s13244-020-00887-2 PMC7423816

[124] Elmahdy M , Sebro R . Radiomics analysis in medical imaging research. J Med Radiat Sci. 2023;70(1):3‐7. 10.1002/jmrs.662 PMC9977659 36762402

[125] Wang T , Lei Y , Fu Y , et al. A review on medical imaging synthesis using deep learning and its clinical applications. J Appl Clin Med Phys. 2021;22(1):11‐36. 10.1002/acm2.13121 PMC7856512 33305538

[126] Shelhamer E , Long J , Darrell T . Fully convolutional networks for semantic segmentation. IEEE Trans Pattern Anal Mach Intell. 2017;39(4):640‐651. 27244717 10.1109/TPAMI.2016.2572683

[127] Shin HC , Roth HR , Gao M , et al. Deep convolutional neural networks for computer‐aided detection: cNN architectures, dataset characteristics and transfer learning. IEEE Trans Med Imaging. 2016;35(5):1285‐1298. 26886976 10.1109/TMI.2016.2528162 PMC4890616

[128] Goodfellow IJ , Pouget‐Abadie J , Mirza M , et al. Generative adversarial nets. Proceedings of the 27th International Conference on Neural Information Processing Systems. 2014;2:2672‐2680.

[129] Liu X , Geng LS , Huang D , Cai J , Yang R . Deep learning‐based target tracking with X‐ray images for radiotherapy: a narrative review. Quant Imaging Med Surg. 2024;14(3):2671‐2692. 38545053 10.21037/qims-23-1489 PMC10963821

[130] Lei Y , Tian Z , Wang T , et al. Deep learning‐based fast volumetric imaging using kV and MV projection images for lung cancer radiotherapy: a feasibility study. Med Phys. 2023;50(9):5518‐5527. 36939395 10.1002/mp.16377 PMC10509310

[131] Zhang Y , Dai X , Tian Z , et al. Landmark tracking in liver US images using cascade convolutional neural networks with long short‐term memory. Meas Sci Technol. 2023;34(5):054002. 36743834 10.1088/1361-6501/acb5b3 PMC9893725

[132] Fung AY , Grimm SY , Wong JR , Uematsu M . Computed tomography localization of radiation treatment delivery versus conventional localization with bony landmarks. J Appl Clin Med Phys. 2003;4(2):112‐119. 12777145 10.1120/jacmp.v4i2.2525 PMC5724476

[133] van Timmeren JE , Leijenaar RTH , van Elmpt W , et al. Survival prediction of non‐small cell lung cancer patients using radiomics analyses of cone‐beam CT images. Radiother Oncol. 2017;123(3):363‐369. 28506693 10.1016/j.radonc.2017.04.016

[134] Korreman SS . Image‐guided radiotherapy and motion management in lung cancer. Br J Radiol. 2015;88(1051):20150100. 25955231 10.1259/bjr.20150100 PMC4628536

[135] O'Neill AG , Jain S , Hounsell AR , O'Sullivan JM . Fiducial marker guided prostate radiotherapy: a review. Br J Radiol. 2016;89(1068):20160296. 27585736 10.1259/bjr.20160296 PMC5604907

[136] Khullar K , Dhawan ST , Nosher J , Jabbour SK . Fiducial marker migration following computed tomography‐guided placement in the liver: a case report. AME Case Rep. 2021;5:15. 33912804 10.21037/acr-20-153 PMC8060155

[137] Klibanov AL , Hossack JA . Ultrasound in radiology: from anatomic, functional, molecular imaging to drug delivery and image‐guided therapy. Invest Radiol. 2015;50(9):657‐670. 26200224 10.1097/RLI.0000000000000188 PMC4580624

[138] Paganelli C , Seregni M , Fattori G , et al. Magnetic resonance imaging‐guided versus surrogate‐based motion tracking in liver radiation therapy: a prospective comparative study. Int J Radiat Oncol Biol Phys. 2015;91(4):840‐848. 25752399 10.1016/j.ijrobp.2014.12.013

[139] Zhao W , Shen L , Islam MT , et al. Artificial intelligence in image‐guided radiotherapy: a review of treatment target localization. Quant Imaging Med Surg. 2021;11(12):4881‐4894. 34888196 10.21037/qims-21-199 PMC8611462

[140] Keall PJ , Sawant A , Berbeco RI , et al. AAPM Task Group 264: the safe clinical implementation of MLC tracking in radiotherapy. Med Phys. 2021;48(5):e44‐e64. 33260251 10.1002/mp.14625

[141] Chen L , Bai S , Li G , et al. Accuracy of real‐time respiratory motion tracking and time delay of gating radiotherapy based on optical surface imaging technique. Radiat Oncol. 2020;15(1):170. 32650819 10.1186/s13014-020-01611-6 PMC7350729

[142] Teuwen J , Gouw ZAR , Sonke JJ . Artificial intelligence for image registration in radiation oncology. Semin Radiat Oncol. 2022;32(4):330‐342. 36202436 10.1016/j.semradonc.2022.06.003

[143] Abbasi S , Mehdizadeh A , Boveiri HR , et al. Unsupervised deep learning registration model for multimodal brain images. J Appl Clin Med Phys. 2023;24(11):e14177. 37823748 10.1002/acm2.14177 PMC10647957

[144] Chai J , Zeng H , Li A , Ngai EWT . Deep learning in computer vision: a critical review of emerging techniques and application scenarios. Machine Learn Appl. 2021;6:100134.

[145] Alzubaidi L , Zhang J , Humaidi AJ , et al. Review of deep learning: concepts, CNN architectures, challenges, applications, future directions. J Big Data. 2021;8(1):53. 33816053 10.1186/s40537-021-00444-8 PMC8010506

[146] Rajput D , Wang WJ , Chen CC . Evaluation of a decided sample size in machine learning applications. BMC Bioinf. 2023;24(1):48. 10.1186/s12859-023-05156-9 PMC9926644 36788550

[147] Chua IS , Gaziel‐Yablowitz M , Korach ZT , et al. Artificial intelligence in oncology: path to implementation. Cancer Med. 2021;10(12):4138‐4149. 33960708 10.1002/cam4.3935 PMC8209596

[148] Ngiam KY , Khor IW . Big data and machine learning algorithms for health‐care delivery. Lancet Oncol. 2019;20(5):e262‐e273. 31044724 10.1016/S1470-2045(19)30149-4

[149] Chalkidou A , O'Doherty MJ , Marsden PK . False discovery rates in PET and CT studies with texture features: a systematic review. PLoS One. 2015;10(5):e0124165. 25938522 10.1371/journal.pone.0124165 PMC4418696

[150] Mongan J , Moy L . Checklist for Artificial Intelligence in Medical Imaging (CLAIM): a guide for authors and reviewers. Radiol Artif Intell. 2020;2(2):e200029. 33937821 10.1148/ryai.2020200029 PMC8017414

[151] Shaikhina T , Khovanova NA . Handling limited datasets with neural networks in medical applications: a small‐data approach. Artif Intell Med. 2017;75:51‐63. 28363456 10.1016/j.artmed.2016.12.003

[152] Vabalas A , Gowen E , Poliakoff E , Casson AJ . Machine learning algorithm validation with a limited sample size. PLoS One. 2019;14(11):e0224365. 31697686 10.1371/journal.pone.0224365 PMC6837442

[153] Cohen J . Statistical Power Analysis for the Behavioral Sciences. 2nd ed. L. Erlbaum Associates; 1988:567.

[154] Shorten C , Khoshgoftaar TM . A survey on image data augmentation for deep learning. J Big Data. 2019;6(1):60. 10.1186/s40537-021-00492-0 PMC8287113 34306963

[155] Mustafa Z , Nsour H . Using computer vision techniques to automatically detect abnormalities in chest X‐rays. Diagnostics (Basel). 2023;13(18):2979. 37761345 10.3390/diagnostics13182979 PMC10530162

[156] Sivanesan U , Wu K , McInnes MDF , Dhindsa K , Salehi F , van der Pol CB . Checklist for artificial intelligence in medical imaging reporting adherence in peer‐reviewed and preprint manuscripts with the highest altmetric attention scores: a meta‐research study. Can Assoc Radiol J. 2023;74(2):334‐342. 36301600 10.1177/08465371221134056

[157] Belue MJ , Harmon SA , Lay NS , et al. The low rate of adherence to checklist for artificial intelligence in medical imaging criteria among published prostate MRI artificial intelligence algorithms. J Am Coll Radiol. 2023;20(2):134‐145. 35922018 10.1016/j.jacr.2022.05.022 PMC9887098

